# Machine Learning Chemical Guidelines for Engineering Electronic Structures in Half-Heusler Thermoelectric Materials

**DOI:** 10.34133/2020/6375171

**Published:** 2020-04-22

**Authors:** Maxwell T. Dylla, Alexander Dunn, Shashwat Anand, Anubhav Jain, G. Jeffrey Snyder

**Affiliations:** ^1^Department of Materials Science and Engineering, Northwestern University, IL 60208, USA; ^2^Department of Materials Science and Engineering, UC Berkeley, CA 94720, USA; ^3^Lawrence Berkeley National Laboratory, Energy Technologies Area, CA 94720, USA

## Abstract

Half-Heusler materials are strong candidates for thermoelectric applications due to their high weighted mobilities and power factors, which is known to be correlated to valley degeneracy in the electronic band structure. However, there are over 50 known semiconducting half-Heusler phases, and it is not clear how the chemical composition affects the electronic structure. While all the n-type electronic structures have their conduction band minimum at either the Γ- or *X*-point, there is more diversity in the p-type electronic structures, and the valence band maximum can be at either the Γ-, *L*-, or *W*-point. Here, we use high throughput computation and machine learning to compare the valence bands of known half-Heusler compounds and discover new chemical guidelines for promoting the highly degenerate *W*-point to the valence band maximum. We do this by constructing an “orbital phase diagram” to cluster the variety of electronic structures expressed by these phases into groups, based on the atomic orbitals that contribute most to their valence bands. Then, with the aid of machine learning, we develop new chemical rules that predict the location of the valence band maximum in each of the phases. These rules can be used to engineer band structures with band convergence and high valley degeneracy.

## 1. Introduction

High thermoelectric performance requires a high thermoelectric quality factor which is proportional to the weighted mobility, *μ*_W_, divided by the lattice thermal conductivity, *κ*_L_ [1]. High weighted mobility, which is correlated to high peak power factor, makes p-type half-Heusler materials strong candidates for thermoelectric applications. These materials owe their high weighted mobilities and high power factors to weak electron-phonon coupling and high valley degeneracy imposed by the symmetry of the Brillouin zone [2–6]. However, there are over 50 known semiconducting half-Heusler compounds [7], and it is not clear how the chemical composition affects the electronic structure. In recent work, machine learning has become a powerful tool for engineering complex properties in cases where the known physical trends are exhausted, but there are many features left to understand [8–13]. Simple models, driven by domain knowledge, are especially useful for discovering ways to engineer these properties, even when there are small amounts of available data [14, 15]. In this work, we use machine learning to develop simple models that explain the electronic structures of half-Heusler phases.

**Figure 1:**
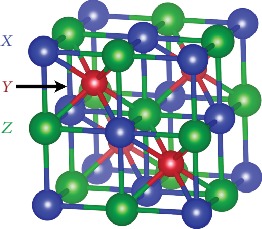
There are three crystallographic sites in the half-Heusler structure: *X* (blue), *Y* (red), and *Z* (green). The *Y*-site is in a body-centered-cubic coordination environment formed by the *X*- and *Z*-sites. The *X*- and *Z*-sites are in tetrahedral coordination environments formed by the *Y*-sites (in the first nearest-neighbor shell) and octahedral coordination environments formed by *X*- and *Z*-sites (in the second nearest-neighbor shell).

To begin to understand electronic structure in the half-Heusler family, we calculated the electronic structures' semiconducting (18 valence electrons) phases using density functional theory (DFT). We chose stable phases reported in the Inorganic Crystal Structure Database (ICSD) alongside 10 phases predicted stable (see Methods) in previous studies from DFT calculations [3, 16, 17]. To quantitatively compare the calculated phases, we decomposed their near band-edge electronic structures into their chemical components—atomic orbitals. For domain experts, atomic orbitals are a powerful basis for interpreting electronic structure [18–21]. For example, small variations in orbital character (*s*/*p*/*d*) explain whether diamond-like semiconductors have direct or indirect band gaps [22]. Based on a chemical map of each phase's atomic orbitals, we find that there are three distinct classes of electronic structures in the half-Heusler family. While all have conduction band minimum at either the *X*-point or the Γ-point—there is more variance in the valence bands—the valence band maximum can be at one of three *k*-points in the Brillouin zone. Phases that are intermediates of the extreme cases even have increased valley degeneracy from the energy convergence of multiple *k*-points at the valence band edge. We use machine learning to elucidate how composition affects the relative energies of these *k*-points, which can direct efforts to engineer band structures with high degeneracy and weighted mobility. Similar to the valence balanced rule that predicts the stability of half-Heusler phases [7], we find that a new valence *difference* rule predicts the relative energies of the *k*-points. Instead of considering the total valence electron count (rule for stability), these rules consider the relative valence electron configurations of the elements on each site of the crystal structure (Figure 1).

## 2. Classifying Valence-Band-Edge Electronic Structures

**Figure 2:**
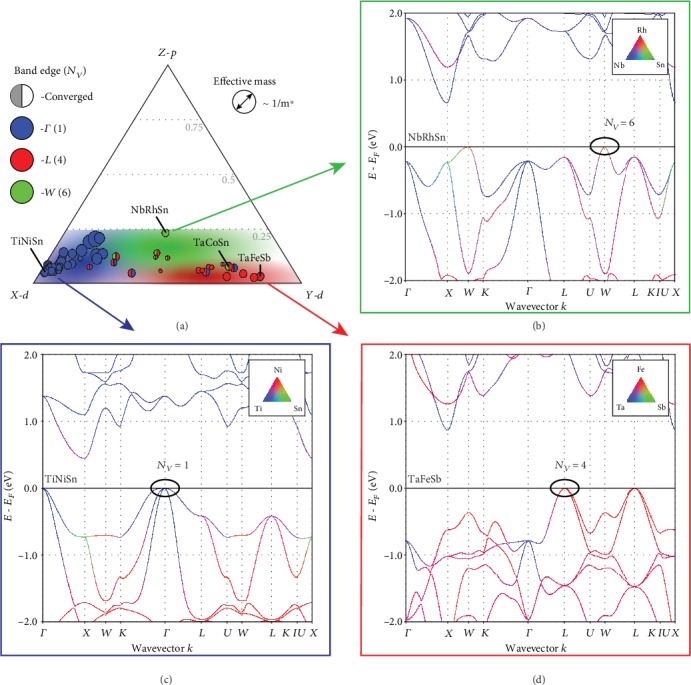
(a) The valence band edges of half-Heusler electronic structures are primarily composed of *d* orbitals from the *X*- and *Y*-sites, and secondarily, *p* orbitals from the *Z*-site. The relative contributions of these basis orbitals describe the type of carrier pockets observed in this structure family. (b) Electronic structures with higher concentrations of *Z*‐*p* orbitals at the band edge have carrier pockets at the *W*-point with high degeneracy. (c) Phases with valence band edges dominated by *X*‐*d* states have carrier pockets at the Γ-point, and (d) band edges dominated by *Y*‐*d* states have carrier pockets at the *L*-point.

When discussing electronic structure in crystalline materials, there are dual aspects to consider. On one hand is the reciprocal space representation—that of electronic band diagrams—where electronic states are indexed by their wave vector, *k*, and band index, *n*. Reciprocal space holds predictive information for many transport properties. For example, materials with low effective mass (*m*^∗^) and high valley degeneracy have favorable electronic properties for thermoelectric applications [23]. However, in this four-dimensional space, it is difficult to study systematic changes in electronic structure with varying chemical composition. The complementary perspective of the electronic structure is represented in real space, where the electronic states correspond to combinations of atomic orbitals [19–21]. Atomic orbitals are the components of electronic structures, analogous to how elements are the components of crystal structures. To further the analogy, relevant portions of the electronic structure are described by atomic orbital *compositions*. In this work, we consider the atomic orbital composition of the valence band edge using the projected density of states [24, 25]. The electronic structures are computed using density functional theory with the PBE functional without accounting for spin-orbit coupling effects. We evaluate the fractions of states that would be occupied by holes in the valence bands (see Methods). This composition depends on the electron chemical potential (Fermi level) and temperature, but for consistency across multiple p-type phases, standard conditions were chosen. In this work, the Fermi level is placed at the valence band edge and the temperature is 700 K, which is near the temperature at the experimental peak power factor for half-Heusler materials [3, 4]. Between the three crystallographic sites (*X*/*Y*/*Z*) and three orbital characters (*s*/*p*/*d*), there are nine components to consider. However, only several of the components contribute meaningfully to the valence states, and 97% of the variation in an orbital character is accounted for by the *X*‐*d*, *Y*‐*d*, and *Z*‐*p* components alone (Figure S2 and Table S1). Therefore, the phases can be represented in a Gibbs phase triangle (Figure 2(a)). In contrast to a conventional phase diagram, which represents the stable phases within a composition region, the “orbital phase diagram” represents the diverse electronic structures expressed by phases within a structure family.

There are three emergent classes of valence band electronic structures (indicated by blue, red, and green). The first class of electronic structure (blue) has the valence band maximum at Γ, which has a degeneracy of one in the first Brillouin zone (*N*_*v*_*k*__). To clarify, we are considering the degeneracy imposed by the symmetry of the Brillouin zone, which does not include the number of degenerate bands (*N*_*v*_*o*__, orbital degeneracy) at that *k*-point (*N*_*v*_ = *N*_*v*_*k*__ · *N*_*v*_*o*__). TiNiSn is an example compound from this class, where the valence band edge is dominated by Ti-*d* states (Figure 2(c)). The second electronic structure class (red) has its valence band maximum at the *L*-point—a degeneracy of four. TaFeSb exemplifies this class, where the band-edge states are dominated by Fe-*d* (Figure 2(d)). In the last class of electronic structure (green), the valence band maximum is at the *W*-point (degeneracy of six). These electronic structures (*e*.*g*., NbRhSn in Figure 2(b)) have relatively higher band-edge contributions from *Z*‐*p* orbitals, which originates from the states along the *X*‐*W* path (green-orange hue). Each of the other electronic structures are hybrids of the three classes. For example, NbCoSn is a hybrid between the *W*-point (green) and *L*-point (red) extremes, with both carrier pockets within 100 meV of the band edge. Irrespective of the electronic structure class, the type of atomic orbitals contributing to each *k*-point (within the first valence band) is similar among all of the half-Heusler materials—the Γ-point is dominated by *X*‐*d* states, the *L*-point is dominated by *Y*‐*d* states, and *Z*‐*p* states are mixed into the *X*‐*W* path. Therefore, the chemical bonding is similar among all the materials. The primary source of variance among their electronic structures is the relative energies of the Γ-, *L*-, and *W*-points, which are linked to the relative energies of their constituent atomic orbitals.

## 3. Valence Difference Rules for Engineering Γ‐*L* Carrier Pockets

**Figure 3:**
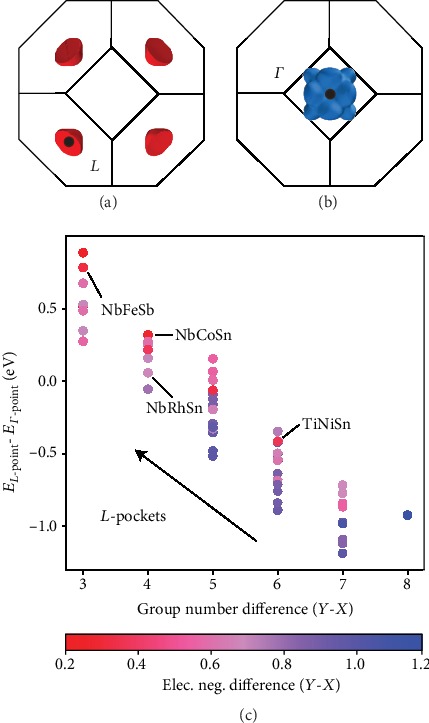
(a) Engineering the relative energies of the *L*-point and (b) the Γ-point controls the degeneracy of half-Heusler materials. (c) The difference in valence electron configuration (encoded in group number) and electronegativity of the *X*- and *Y*-species determines the energy offset of these *k*-points.

Engineering the Γ‐*L* energy offset tunes the valley degeneracy and the thermoelectric performance of half-Heusler materials [4]. The relative energies of the Γ- and *L*-points are described by simple, chemical differences between the *X*- and *Y*-species. The dominant, first-order effect is the difference in valence between the *X*- and *Y*-species, which is encoded in their group (column) number on the periodic table. In a linear model, differences in valence account for over 85% of the variation in the Γ‐*L* energy offset (Figure 3). Compounds with larger differences in valence have valence band maxima at Γ (*e.g.*, TiNiSn, where Ni has six more valence electrons than Ti), while compounds with smaller differences in valence have valence band maxima at *L* (*e.g.*, NbFeSb, where Fe has only three more valence electrons than Nb). A second-order descriptor is the difference in Pauling's electronegativity between the *X*- and *Y*-species, which can account for differences in the Γ‐*L* energy offset between compounds with isovalent species (*e.g.*, NbCoSn and NbRhSn). Furthermore, elemental characteristics of the *Z*-species do not improve the prediction of the Γ‐*L* energy offset, likely because the energies are properties of the *X*- and *Y*-species orbitals. Recall that the Γ- and *L*-point states are formed from the *X*‐*d* and *Y*‐*d* orbitals.

The valence difference rule extends beyond the semiconducting phases to metastable phases with 17 and 19 valence electrons [26–28], which are p- and n-type metals (Figure S7). For example, while the energy difference between the Γ- and *L*-points is nearly zero for TiCoSb, the Γ-pocket dominates the valence band maximum in the Ni-substituted analog; TiNiSb has a larger valence difference and 19 valence electrons. Conversely, in the Fe-substituted analog, the *L*-pocket dominates; TiFeSb has 17 valence electrons and a smaller difference in valence. While TiNiSb and TiFeSb are not stable themselves, there are implications for forming solid solutions between TiCoSb and either of the metallic end-members (electronic doping) [29]—the relative energies of the Γ- and *L*-points may change.

## 4. Engineering Highly Degenerate *W*-Pocket Materials

**Figure 4:**
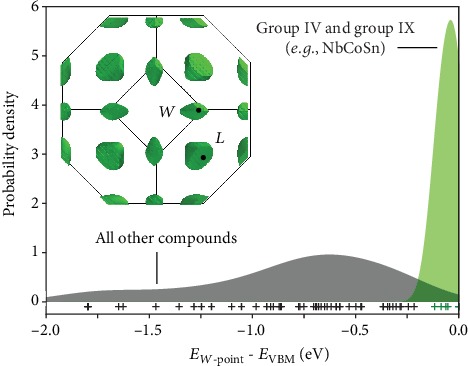
The *W*-point is at or near the valence band maximum for compounds with *both* a group IV element on the *Z*-site (Sn or Ge) and a group IX on the *Y*-site (Co, Rh, or Ir). Furthermore, in six of the seven *W*-pocket materials, the *L*-point is converged within 100 meV of the band edge (total degeneracy of ten).

Materials that contain *both* group IV (*e.g.*, Sn) and group IX (*e.g.*, Co) elements adopt a distinct class of electronic structure, where the *W*-point is at or near the valence band edge (Figure 4). In six of these seven phases, the *W*-point and *L*-point are both within 100 meV of the valence band edge, effectively converged at 1200 K. The exception to the converged cases is NbRhSn, which is the most extreme example of the *W*-pocket class. While only Sn- and Ge-containing end-member phases are reported stable in the literature, the calculation of metastable NbCoPb confirms that this valence rule extends beyond Sn- and Ge-containing compounds (Figure S8). Entropy-stabilized solid solutions between NbCoSn and NbCoPb could benefit from reduced lattice thermal conductivity from alloy scattering and retain valley-high degeneracy throughout the solid solution [30–32]. However, the carrier density must be tuned to optimize the thermoelectric transport properties. There are three sites where aliovalent substitution can introduce additional holes in the system and tune the carrier density. We have computed several site-substituted end-members to investigate the potential changes in band structure induced by candidate dopant elements (Figure S8). Substituting on the *X*- and *Y*-sites has the expected behavior of tuning the Γ‐*L* energy offset, based on the valence difference rules developed in Section 3.

Substituting Ti on the Nb-site (*X*-site) raises the relative energy of the Γ point, since the valence difference between Ti and Co is larger than between Nb and Co. Introducing Fe on the Co-site (*Y*-site) has the opposite effect, and pushes the *L*-point above the *W*-point, unconverging the bands. However, substituting In on the Sn-site (*Z*-site) has an entirely new effect. In NbCoIn, the *X*-point is at the valence band edge. This compound has an entirely different class of electronic structure, distinct from the three archetypal band structures identified in Figure 2. The p states from elemental In appear to promote the *X*-point to the valence band edge. The band structure has a flat and dispersive character between the *X*- and *W*-points, which is similar to the band character found in SrTiO_3_ and some full-Heusler phases [33, 34]. There may be differences in transport properties between materials doped on each of the three sites.

## 5. Conclusions

We have mapped the electronic structures of semiconducting half-Heusler phases according to the atomic orbital composition of their valence bands. This mapping is termed an orbital phase diagram, and it reveals that there are three well-distinguished classes of electronic structures. The *k*-points forming the valence band maximum are different for each electronic structure class. The relative energies of these *k*-points can be controlled using simple rules based on the valence electron configurations of the elemental species. The difference in valence between the *X*- and *Y*-species controls the relative energies of the Γ- and *L*-point energies, while controlling the valence of the *Y*- and *Z*-species can lead to the emergence of highly degenerate carrier pockets at the *W*-point. These rules extend beyond the semiconducting phases, as demonstrated by calculations of metastable 17 and 19 valence electron phases.

These results form a foundation for exploring the space of possible solid solutions in this structure family. Forming solid solutions is incredibly important in the half-Heusler family for suppressing their high lattice thermal conductivities [4, 35–41]. While lattice thermal conductivities in solid solutions are quantitatively described by empirical models [30–32], changes in electronic properties are understood more qualitatively. To the first order, the apparent band structure in a solid solution is a linear interpolation between the end-member electronic structures [42–47]. For example in the Zintl structure family, the band gap and effective mass in n-type Mg_3_Sb_2_Mg_3_Bi_2_ change linearly with composition between Mg_3_Sb_2_ and Mg_3_Bi_2_ [48]. In the III-V semiconductors, the band gap of InAs-GaAs changes linearly as well [49]. In future work on half-Heuslers, the effects of forming solid solutions on the electronic structures could be studied by calculating the backfolded band structures [50, 51] or analyzing their transport properties. Furthermore, the orbital phase diagram technique will be useful for tracking the changes in electronic structure throughout the solid solutions.

## 6. Methods

### 6.1. Calculation Details

Electronic structure calculations were carried out using a plane-wave basis (cutoff energy of 520 eV) in the VASP package with PAW pseudopotentials and the PBE functional [25, 52–54]. Spin-orbit coupling corrections were not applied to these calculations. The structural degrees of freedom were relaxed using 12 × 12 × 12 Monkhorst-Pack *k*-point meshes [55], followed by relaxation of the electronic degrees of freedom using 15 × 15 × 15 meshes. Finally, a non-self-consistent field calculation with 20 × 20 × 20 gamma-centered meshes was used to calculate quantitatively accurate density of states with tetrahedron smearing [56]. In addition, inertial (conductivity) effective masses were calculated using the BoltzTraP package [57]. This set of calculations were performed with the atomate workflow software [58]. The projected density of states and chemical composition were featurized in the matminer package using the SiteDOS and ElementProperty (with pymatgen data) featurizers [59]. The Fermi surfaces of the electronic structures were visualized using the pymatgen package [60]. The most important atomic features for modeling the Γ‐*L* energy offset were determined by ridge regression [61]. The calculations were performed on stable phases reported in the Inorganic Crystal Structure Database (ICSD) alongside 10 phases (HfAsIr, HfBiRh, HfNiPb, HfPdPb, NbSbOs, TaSbOs, TaSnRh, TiAsIr, TiSnPd, and ZrAsIr) predicted stable in previous studies from DFT calculations [3, 16, 17].

### 6.2. Measuring Electronic Structure Compositions

In p-type semiconductors, charge-transporting holes occupy states in the valence bands according to the distribution function for holes (*h* = 1 − *f*, where *f* is the Fermi-Dirac distribution function) [62]. These valence states are ascribed to particular atomic orbitals in the projected density of states (*g*_*i*_). The number of occupied holes from a particular atomic orbital (*p*_*i*_) is accumulated from the valence band states (Figure S1).

(1) $$\begin{array}{c} p_{i}=\int_{\text{VB}}g_{i}\left(E\right)\cdot h\left(E\right)\cdot dE. \end{array}$$

The fractions of atomic-like holes (*x*_*i*_ = *p*_*i*_/*Σ*_*i*_*p*_*i*_) describe the composition of the system of holes in a particular phase. The composition depends on the Fermi level and the temperature. In this work, the Fermi level is placed at the band edge and the temperature is 700 K. When analyzing conduction bands, the Fermi-Dirac distribution function can replace the hole distribution function.

### 6.3. Modeling the Γ‐*L* Energy Offset

Regression was used to identify design principles for engineering the Γ‐*L* energy offset. Fivefold crossvalidation was used to score the trained models according to the coefficient of determination (*r*^2^). The model pipeline consisted of standard scaling of the input features (generated from the ElementProperty featurizer with pymatgen data, which was applied to each crystal site) to zero mean and unit variance, followed by ridge regression trained by gradient descent with early stopping. The model scoring was optimized over a grid of tolerance values for early stopping. The optimized model scores and regression weights were collected for a series of regularization strengths (Figure S4/5). As the regularization penalty was decreased, the *X*- and *Y*-site group number became the most dominant feature as measured by the regression weights. Ordinary least squares reveals that over 85% of the variation in the energy offset is explained by the difference in group number between the *X*- and *Y*-sites alone (Figure S6).

### 6.4. Modeling the *W*-Pocket Class

It was observed that compounds with both group IV (*e.g.*, Sn) and group IX (*e.g.*, Co) elements adopt the *W*-pocket type electronic structure. To confirm that this rule describes the distinct class of electronic structure, we compared the distributions of energy offsets between compounds that follow this chemical rule and those that do not (Figure 4). The distributions were estimated using a Gaussian kernel. It can be seen that the two distributions are distinct.

## Supplementary Materials

Supplementary Materials I. Measuring electronic structure compositions. II. Regression of Γ‐*L* offset. III. Metastable compound calculations. IV. Band structures of the 18 electron half-Heuslers
