# Supplementary material for: Machine Learning Chemical Guidelines for Engineering Electronic Structures in Half-Heusler Thermoelectric Materials
Source: Research (Wash D C). 2020 Apr 22;2020:6375171. doi: 10.34133/2020/6375171 (PMC7193307; doi:10.34133/2020/6375171)
Supplement: Supplementary Materials — I. Measuring electronic structure compositions. II. Regression of Γ‐L offset. III. Metastable compound calculations. IV. Band structures of the 18 electron half-Heuslers [file 6375171.f1.pdf]

Supporting Information

I Measuring Electronic Structure Compositions

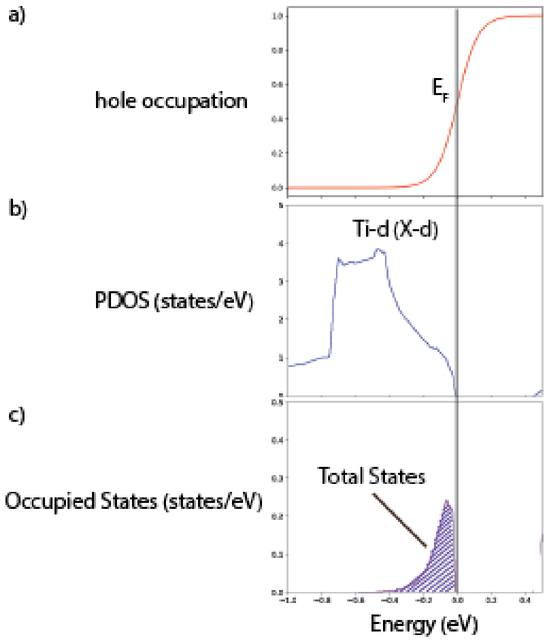

Figure 1: **a)** Holes occupy the valence bands according to the distribution function for holes. **b)** Each projected density of states (from each orbital on each site) is sampled by the occupation function **c)** to determine the total number of holes attributed to a particular atomic orbital. Standard conditions are established to compare different half-Heusler phases. The Fermi-level (E_F) is placed at the valence band maximum and the temperature of the hole distribution is 700 K.

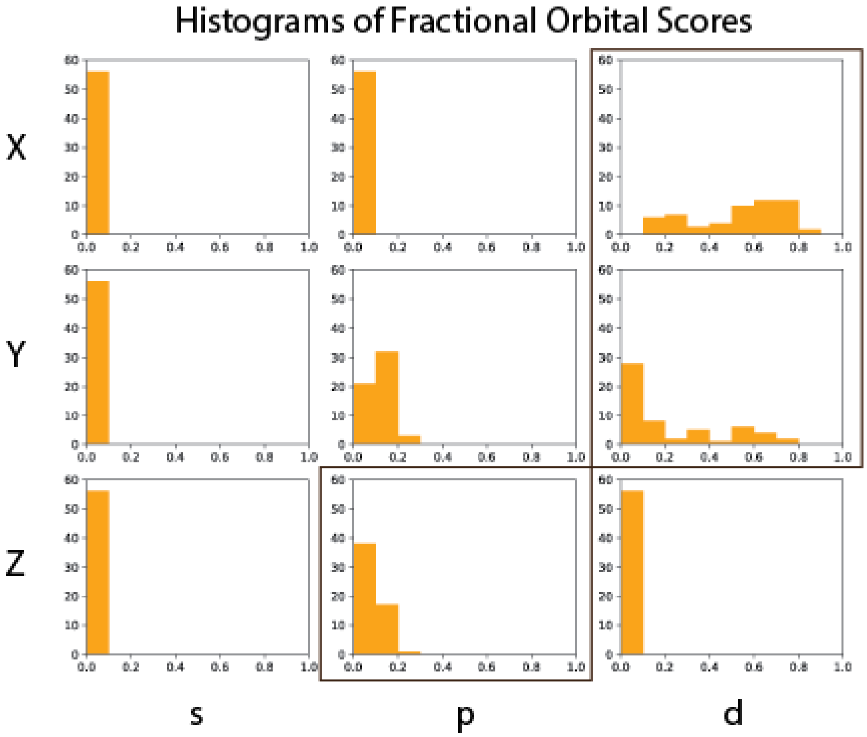

Figure 2: Histograms of the fractional orbital scores computed using the method in Figure S1. The axes for each plot are number of compounds (y-axis) within a particular fractional composition bin (x-axis). Three orbitals (X-d, Y-d, and Z-p) account for over 97% of the variance in orbital characters. Therefore the compounds can be plotted in the Gibbs phase triangle. A similar triangle was plotted using Y-p instead of Z-p, and similar results were found. However, the Z-p scores better differentiated the W-pocket materials from the Γ- and L-pocket materials.

Orbital	Fraction of variance
X-d	0.423
Y-d	0.534
Y-p	0.0203
Z-p	0.0165
Z-d	0.00252
X-p	0.00239
Y-s	9.10e-06
X-s	4.15e-06
Z-s	1.39e-05

Table 1: Fraction of variance from each atomic orbital among all of the calculated electronic structures

II Regression of Γ-L Offset

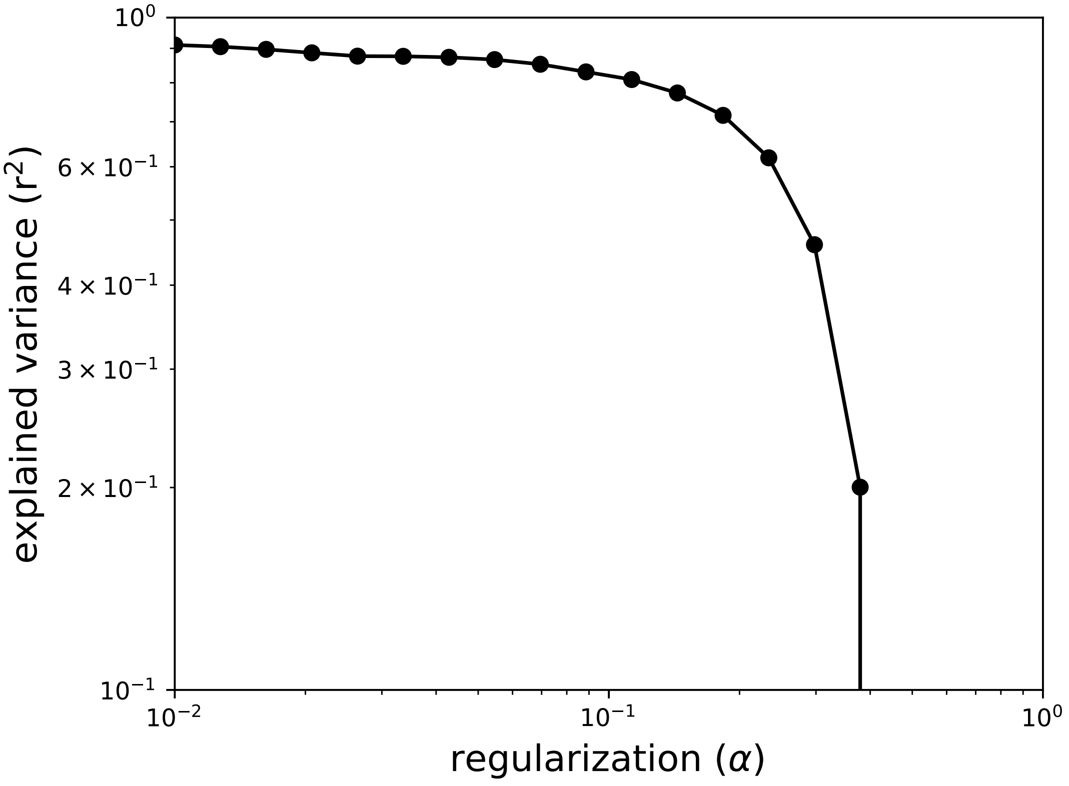

Figure 3: Results of ridge regression in a 5-fold cross-validation loop. Explained variance as a function of regularization. The explained variance starts to plateau around $\alpha = 0.2$.

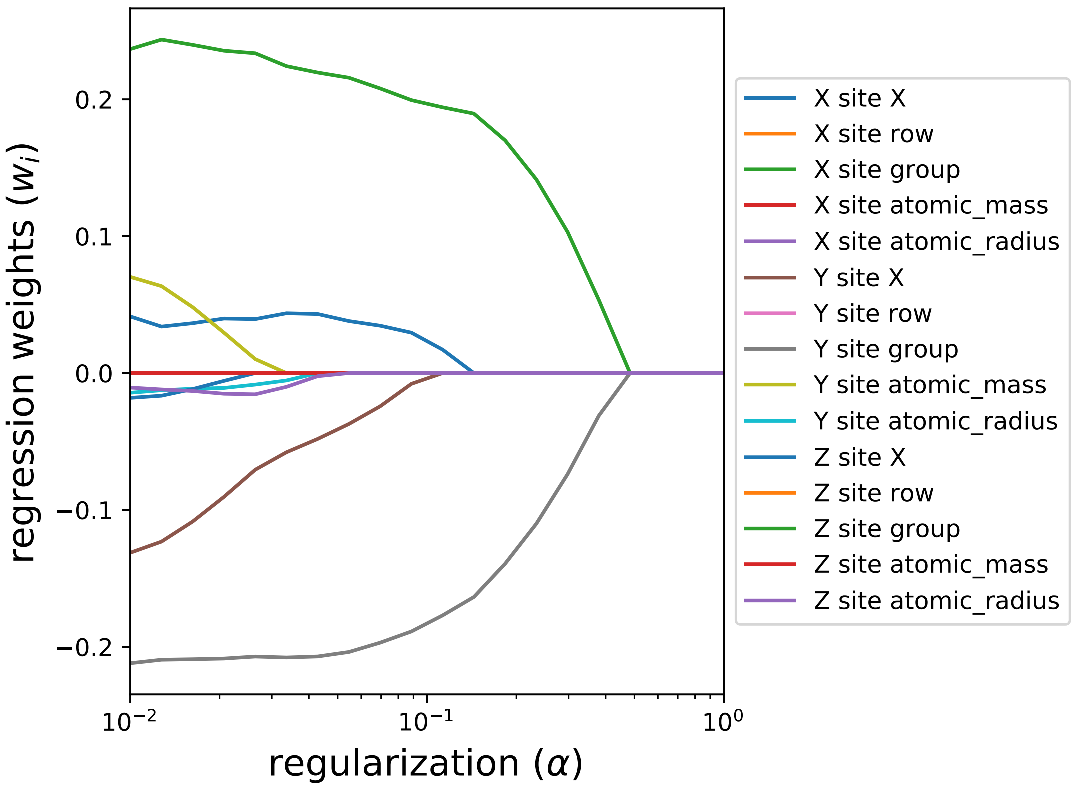

Figure 4: The regression coefficients as a function of regularization. The X-site group number and Y-site group number become the most important features around $\alpha = 0.3$. They are equal and opposite in sign, which suggests that their difference controls the energy offset. The next set of features to appear are the X- and Y-site electronegativity.

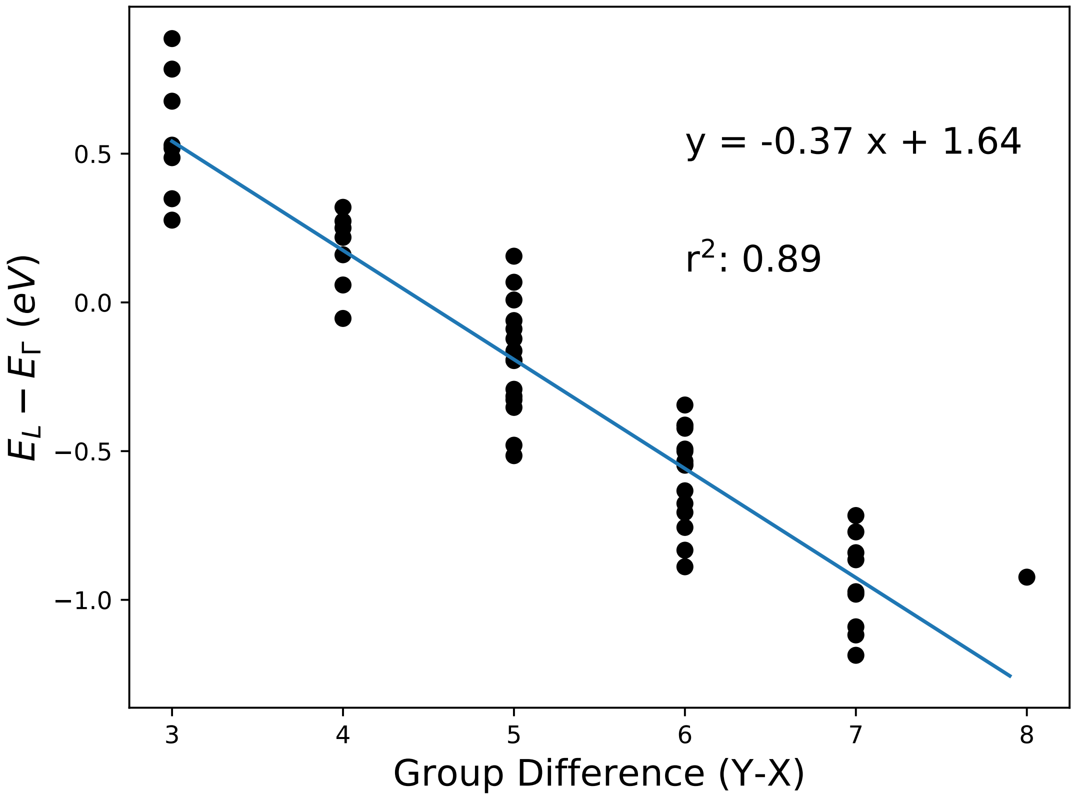

Figure 5: Results of ordinary least squares on the dataset. 89% of the variation in energy difference between the Γ- and L-point is explained by the difference in group number between the Y- and X-species.

III Metastable Compound Calculations

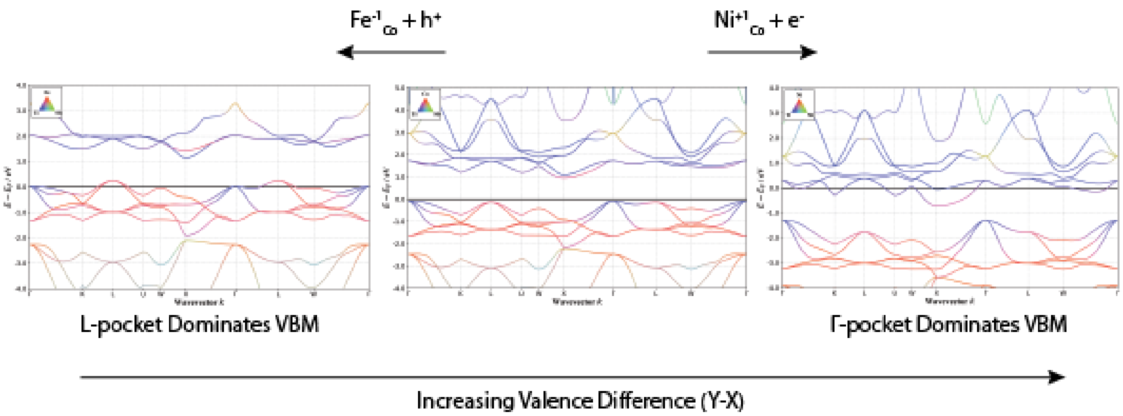

Figure 6: Calculating metastable 17e- and 19e- compounds confirms that valence difference rules control the relative positions of the valence band Γ- and L-energies. Substituting Fe on the Co site of TiCoSb dopes the material p-type and raises the energy of the L-point relative to Γ. Substituting Ni on the Co site dopes the material n-type and raises the energy of the Γ-point relative to the L-point. The relative energies of Γ and L appear to be primarily controlled by the valence difference between the X- and Y-sites and not affected by the location of the Fermi level. This virtual experiment indicates that the effect of dopants on the band structure can be predicted from the valence difference rule.

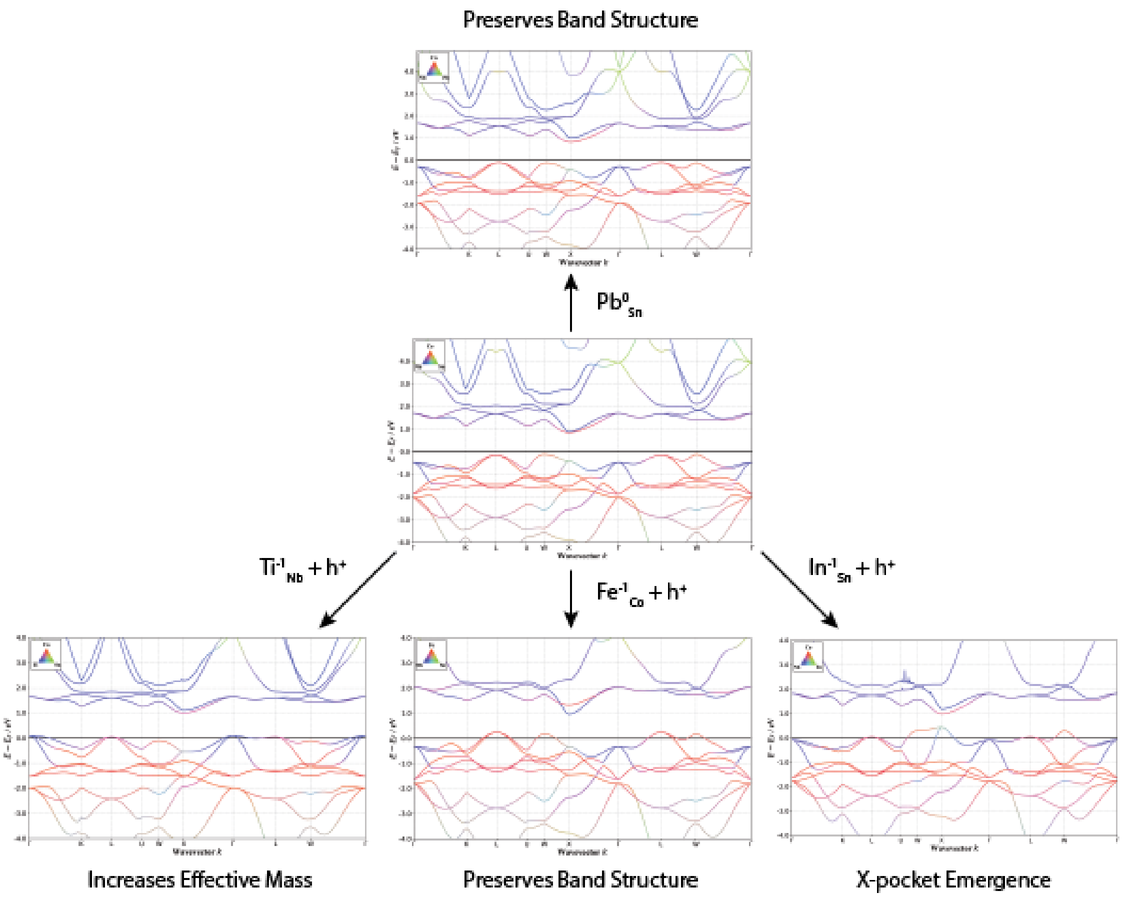

Figure 7: We explore the effects of site-substitution on NbCoSn. The iso-valent substitution of Pb on the Sn site does not significantly affect the band structure. Solid-solutions between the Sn- and Pb-analogs could lower the thermal conductivity through phonon alloy scattering without significantly impacting the electronic structure. P-type, aleo-valent substitutions on each of the different sites have different effects on the electronic structure. Ti on the Nb-site raises the energy of the Γ point per the valence difference rule, and increases the effective mass of the hole-pockets. Substituting Fe on the Co-site raises the energy of the L-point, but has less of an impact on the electronic structure compared to substituting Ti. In on the Sn-site has remarkable changes to the electronic structure, raising the energy of the X-point. This is a class of electronic structure not observed in the stable 18e- materials, so In may be an interesting dopant to consider. Experiments should investigate the effects of different site-dopings on the thermoelectric transport properties.

IV Band Structures of the 18 electron half-Heuslers

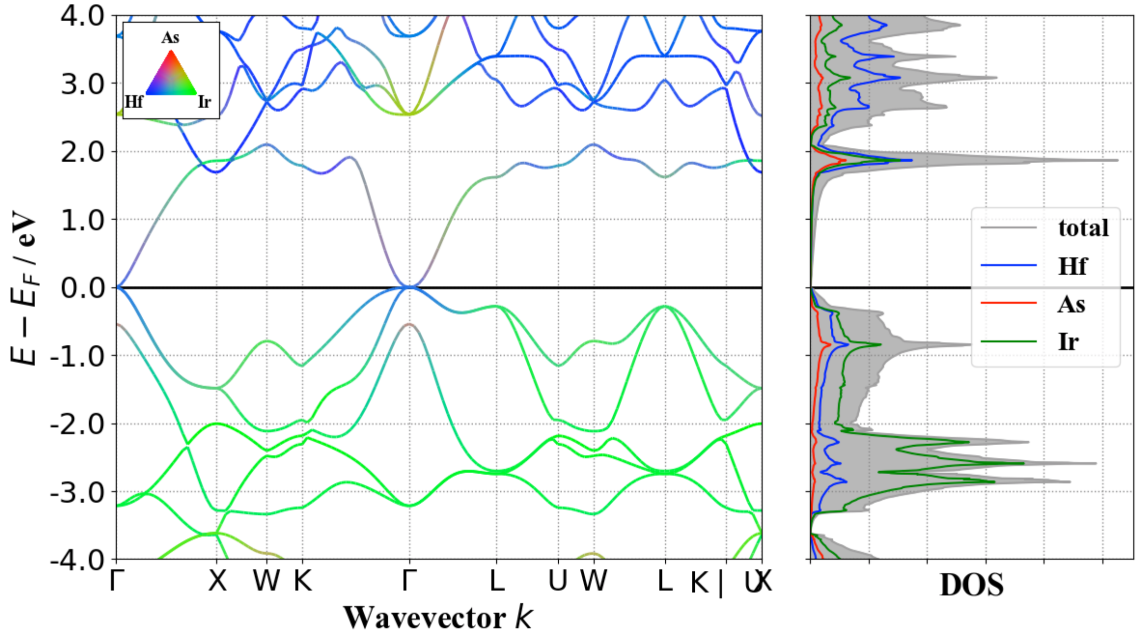

Figure 8: HfAsIr

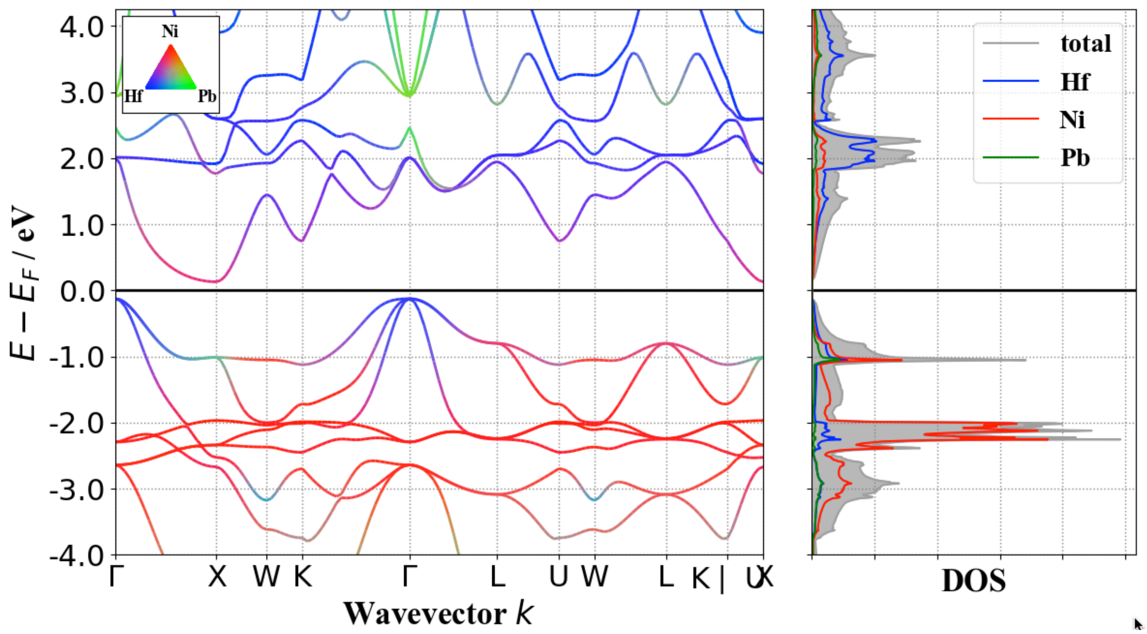

Figure 9: HfNiPb

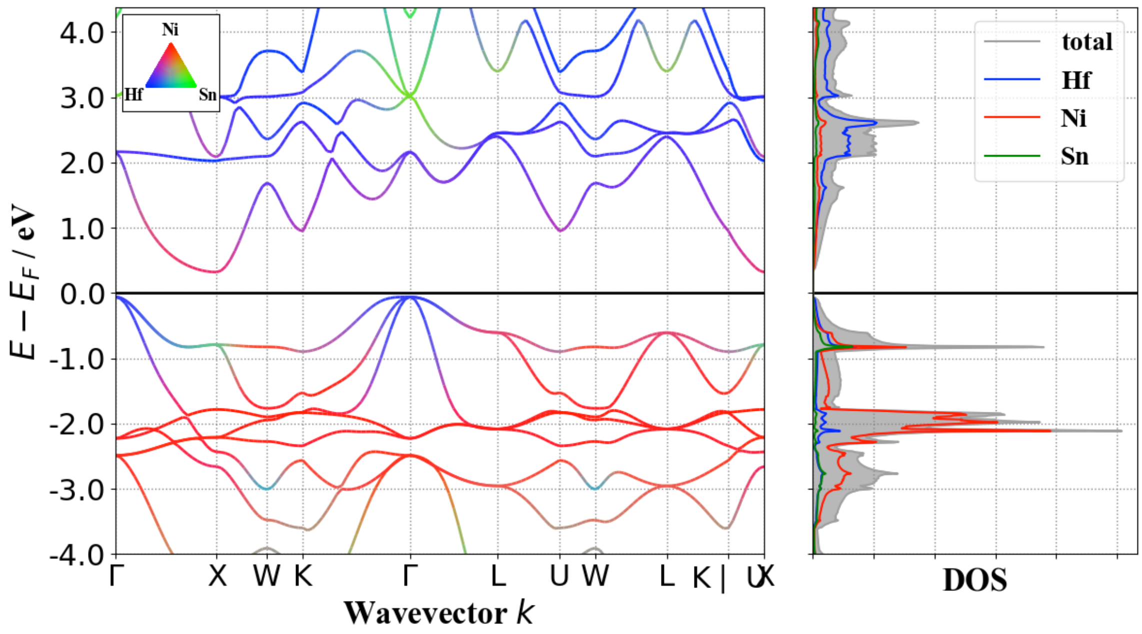

Figure 10: HfNiSn

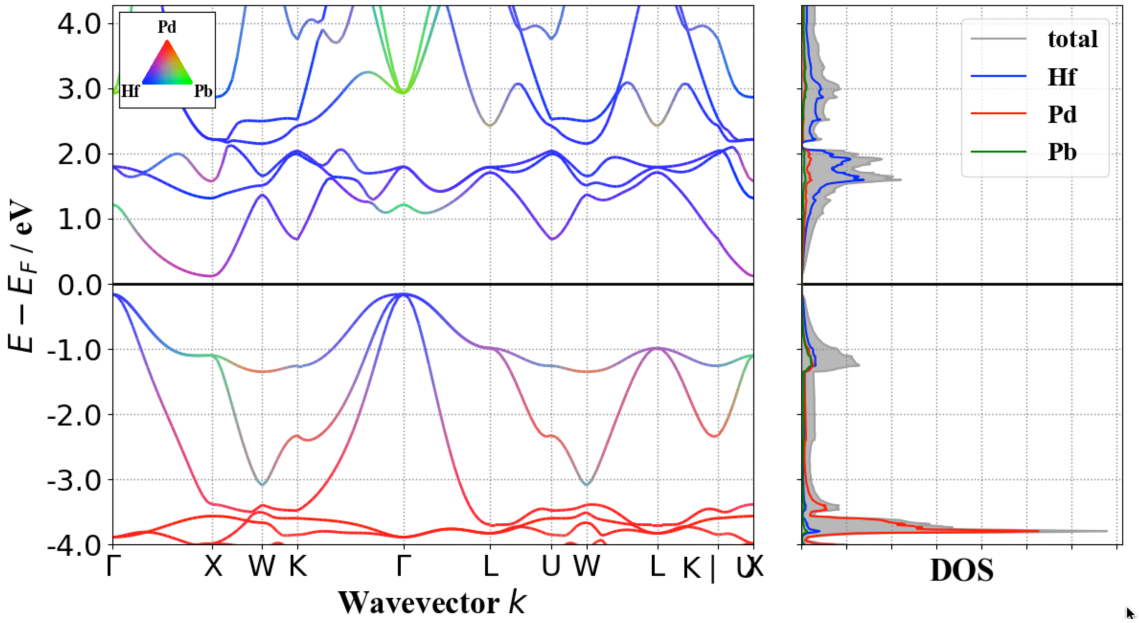

Figure 11: HfPdPb

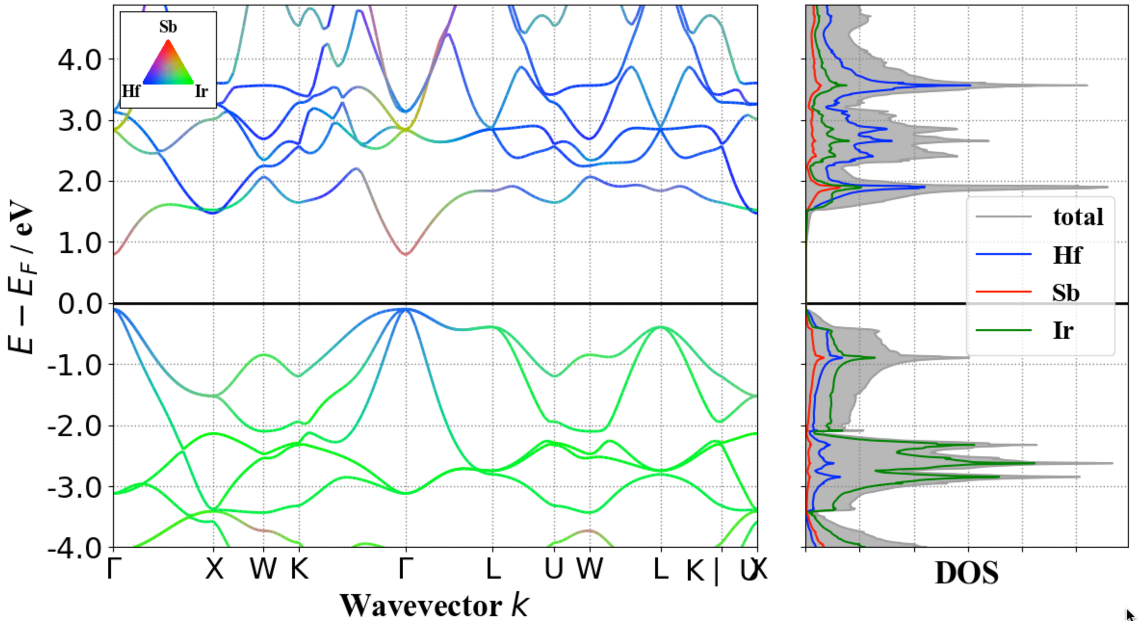

Figure 12: HfSbIr

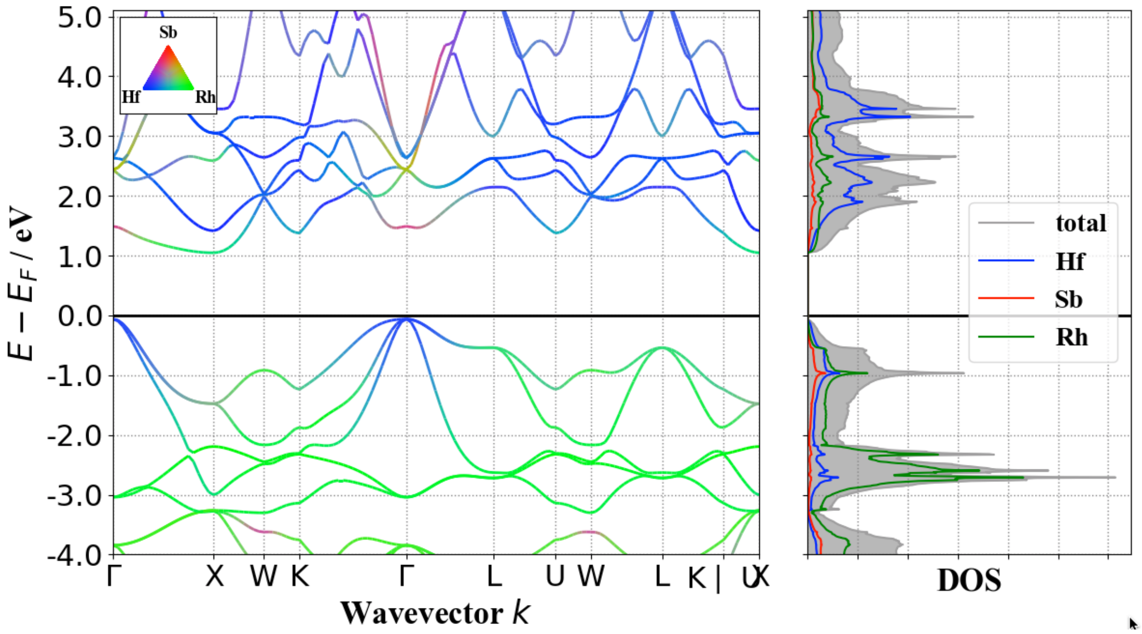

Figure 13: HfSbRh

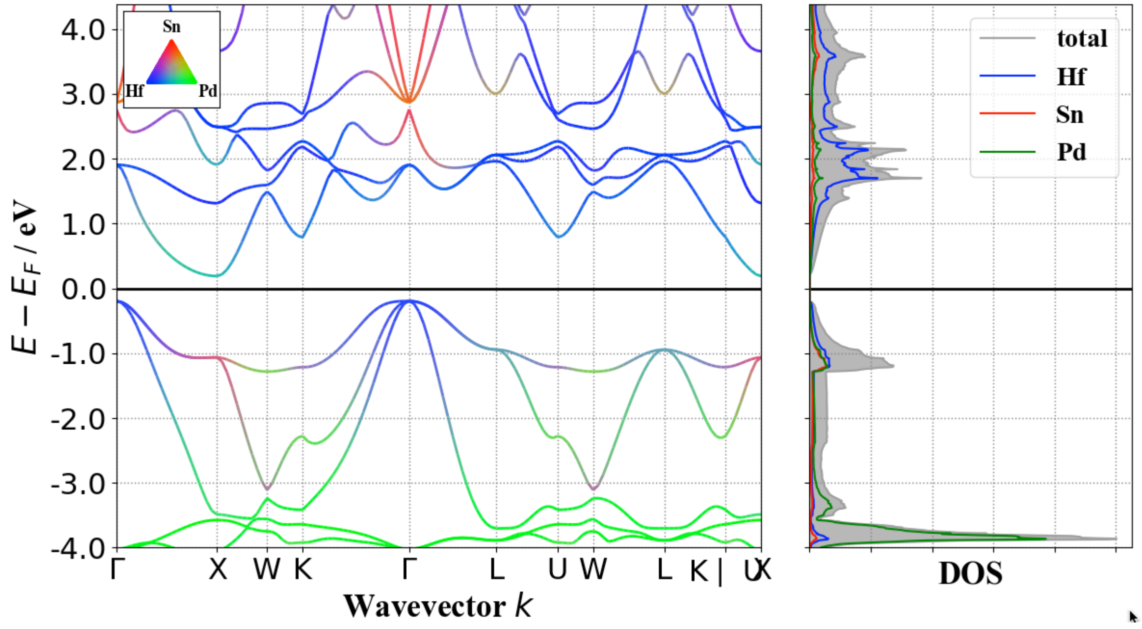

Figure 14: HfSnPd

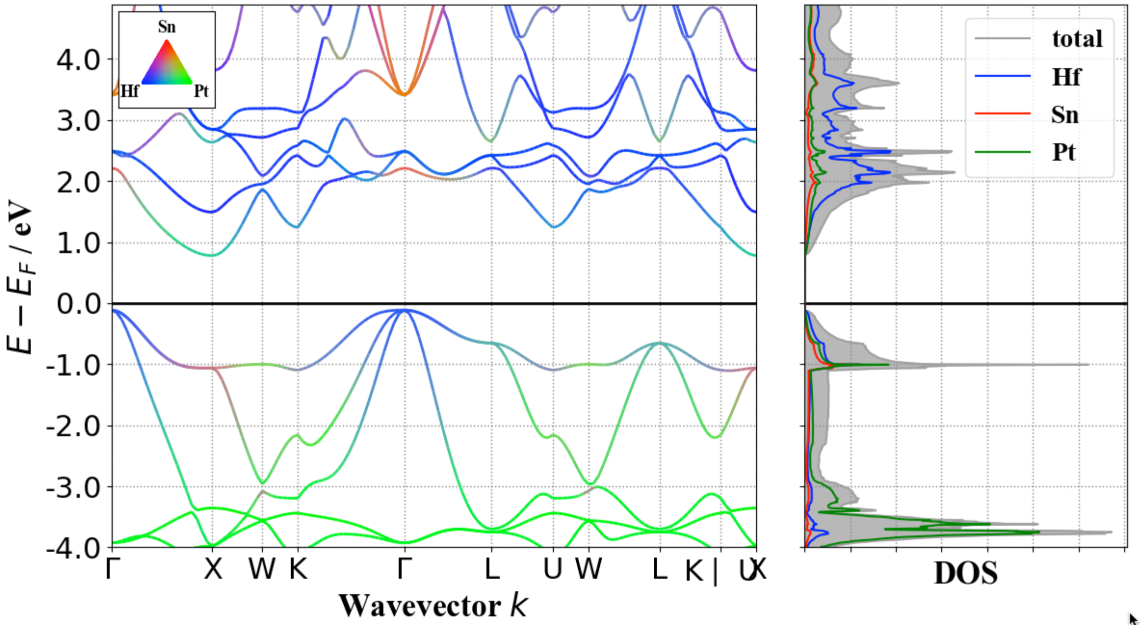

Figure 15: HfSnPt

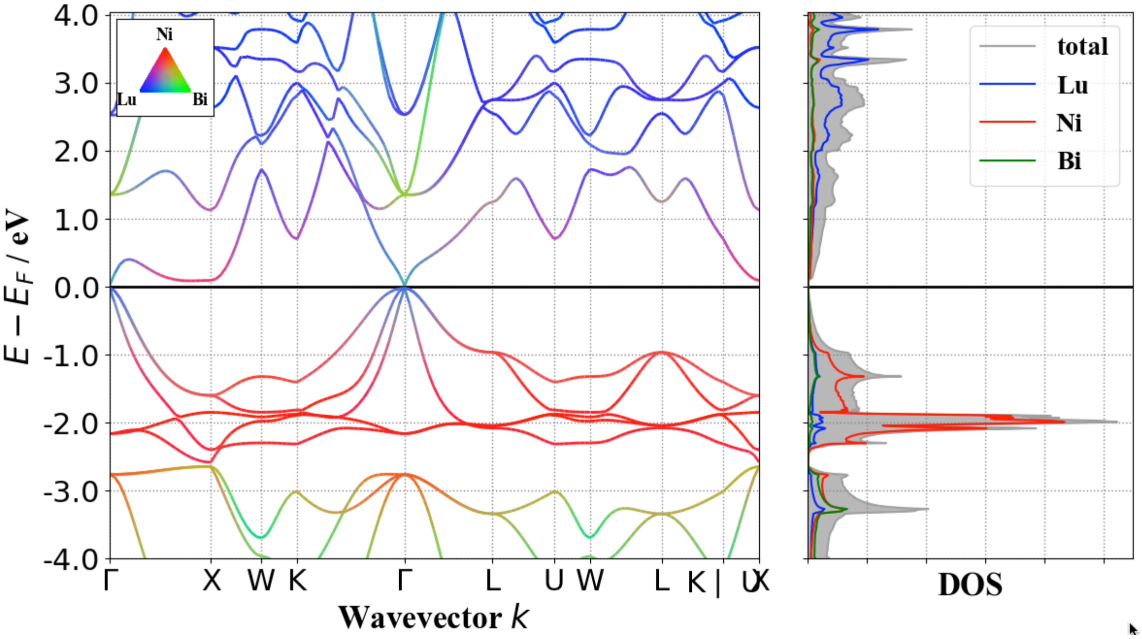

Figure 16: LuNiBi

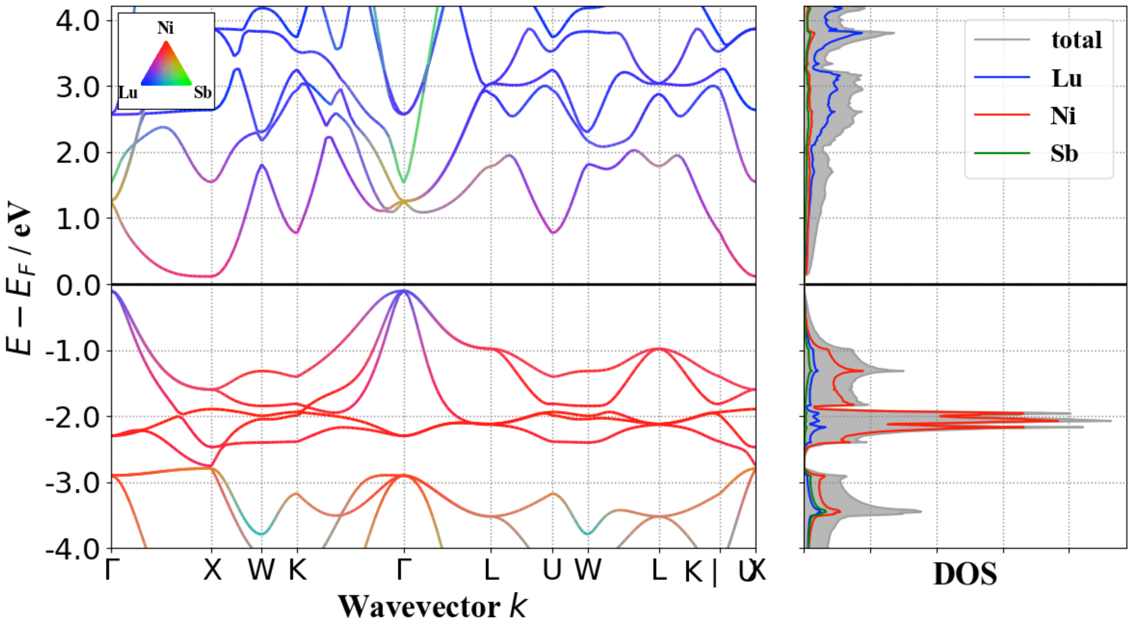

Figure 17: LuNiSb

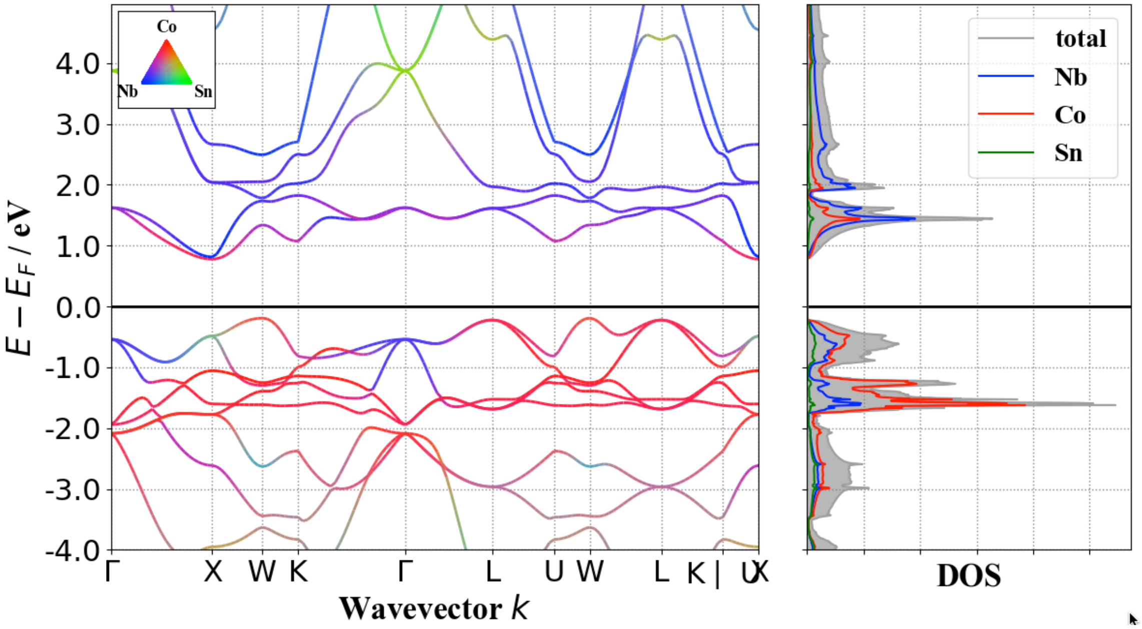

Figure 18: NbCoSn

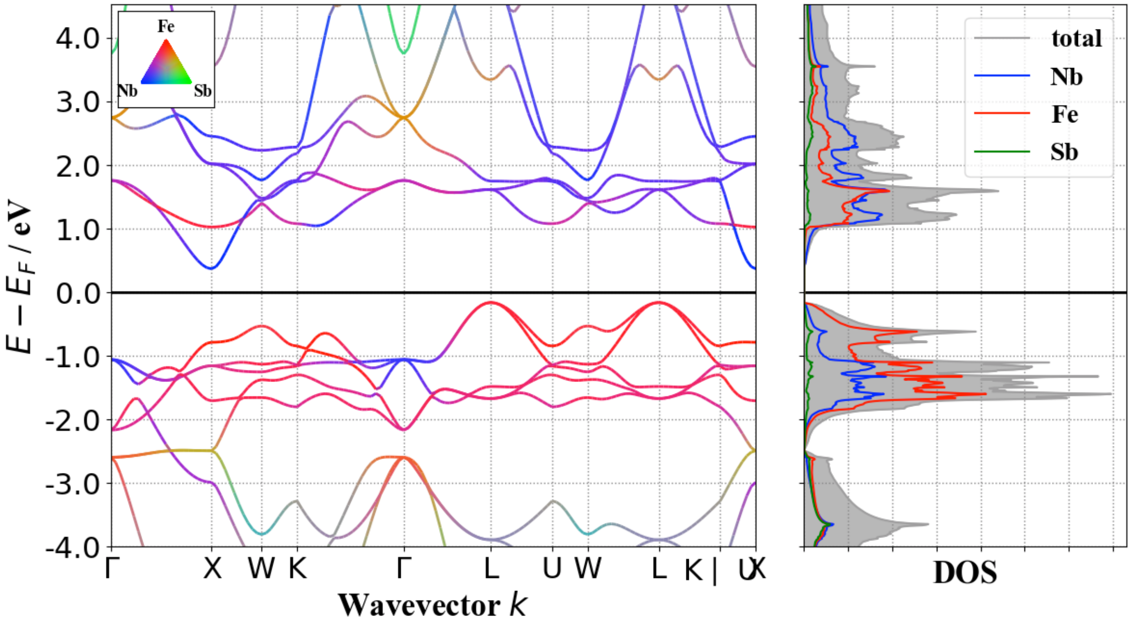

Figure 19: NbFeSb

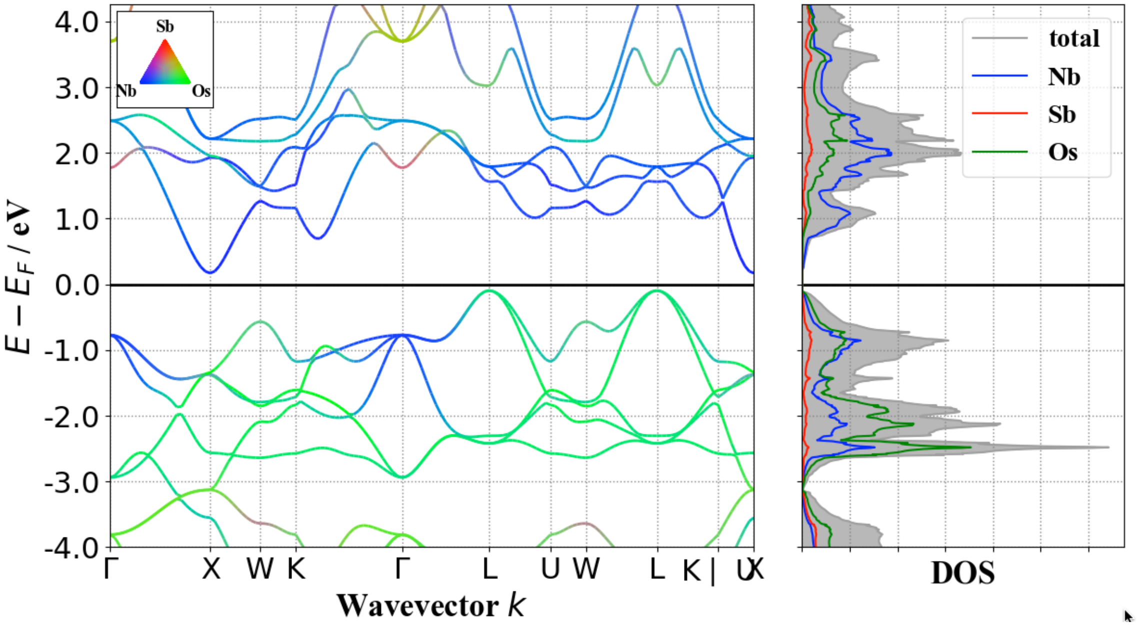

Figure 20: NbSbOs

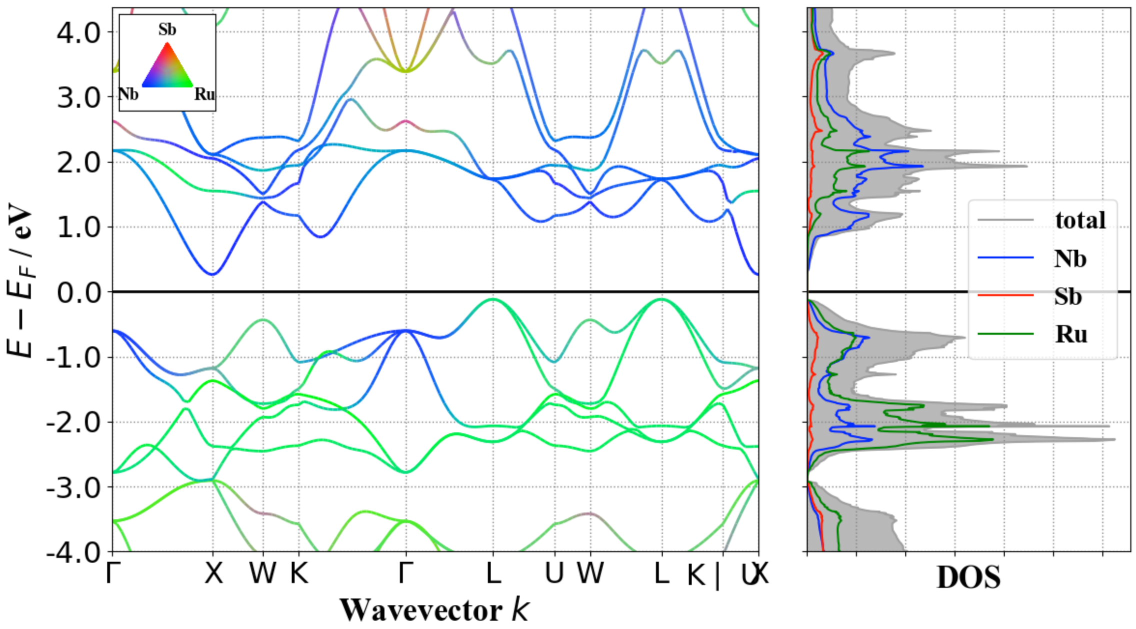

Figure 21: NbSbRu

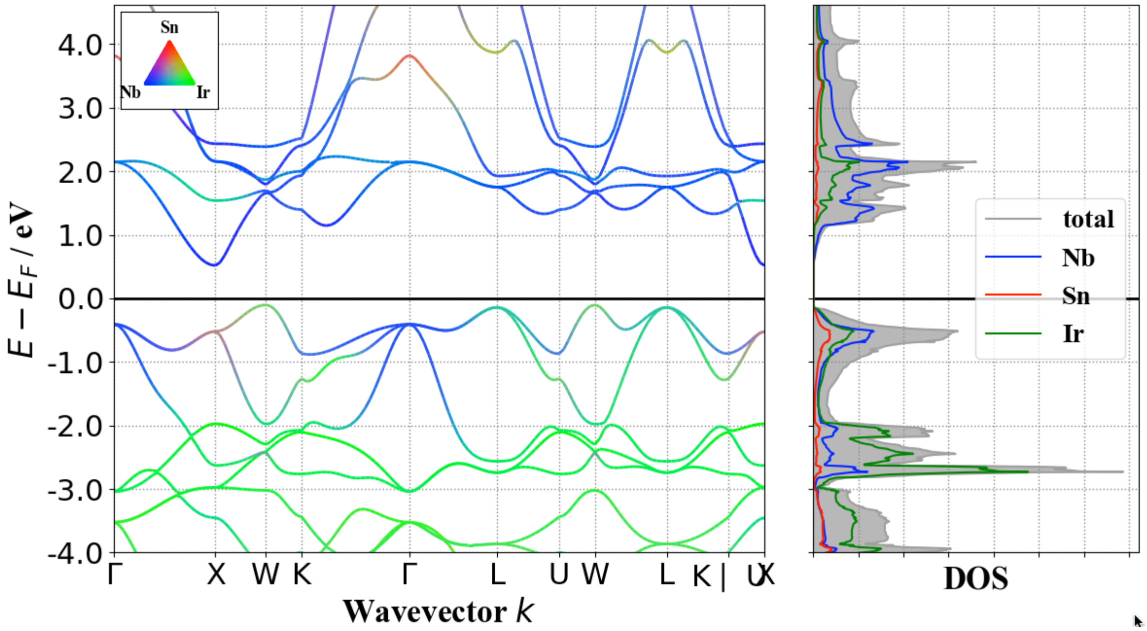

Figure 22: NbSnIr

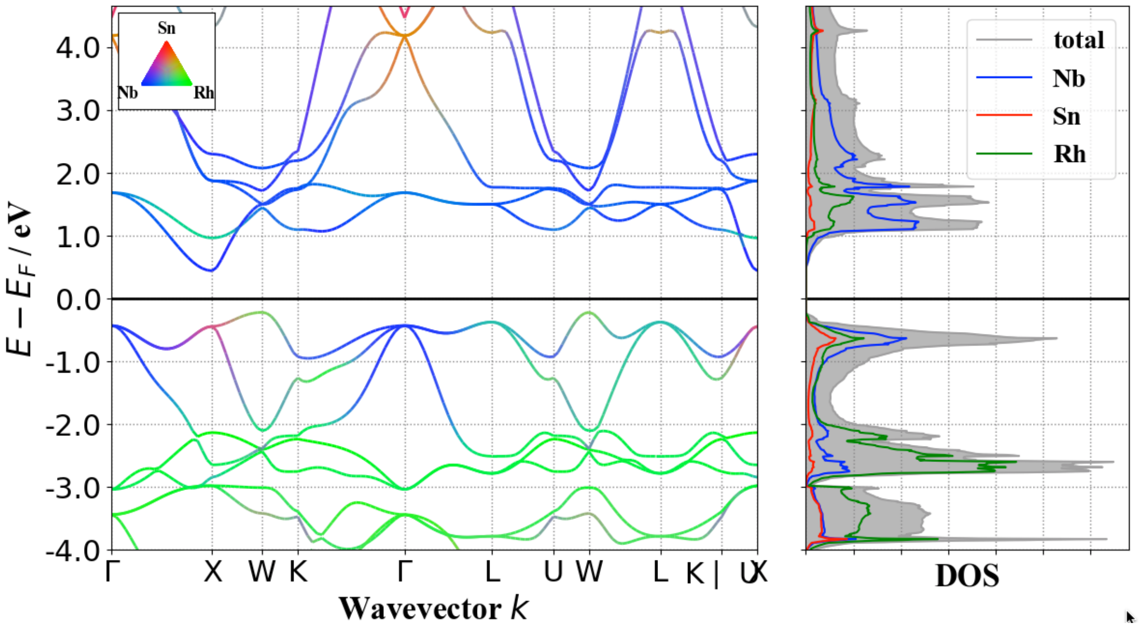

Figure 23: NbSnRh

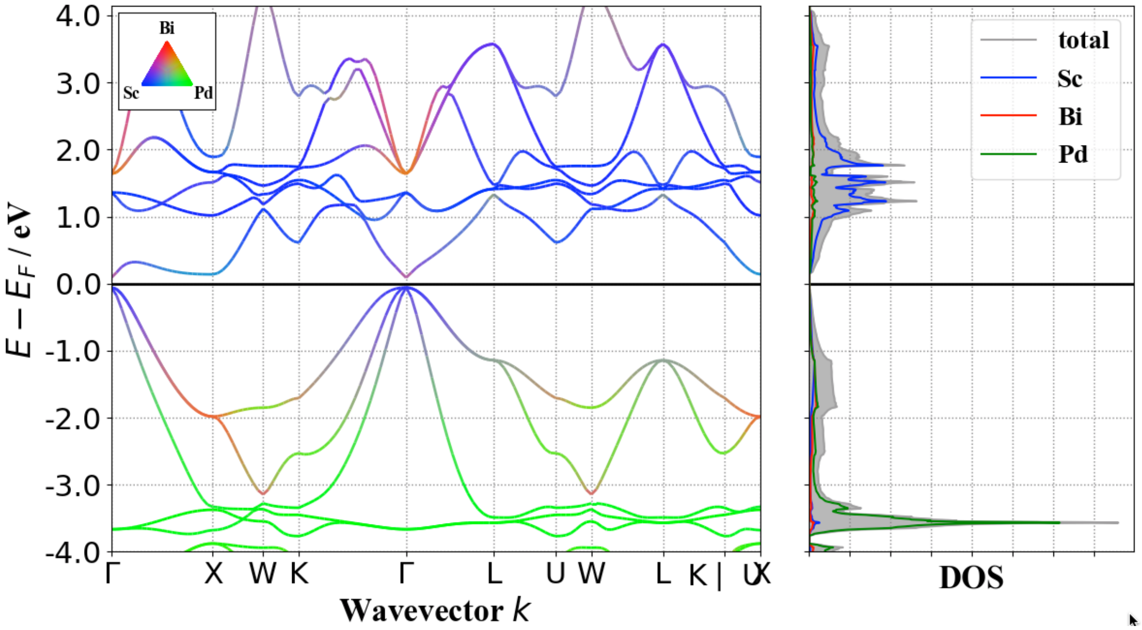

Figure 24: ScBiPd

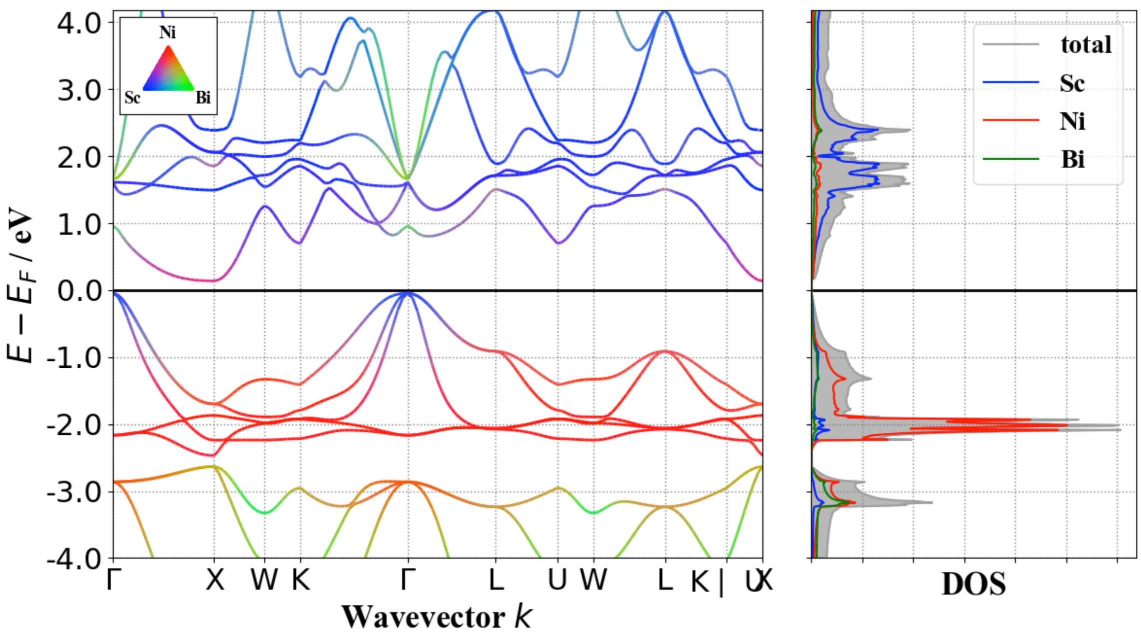

Figure 25: ScNiBi

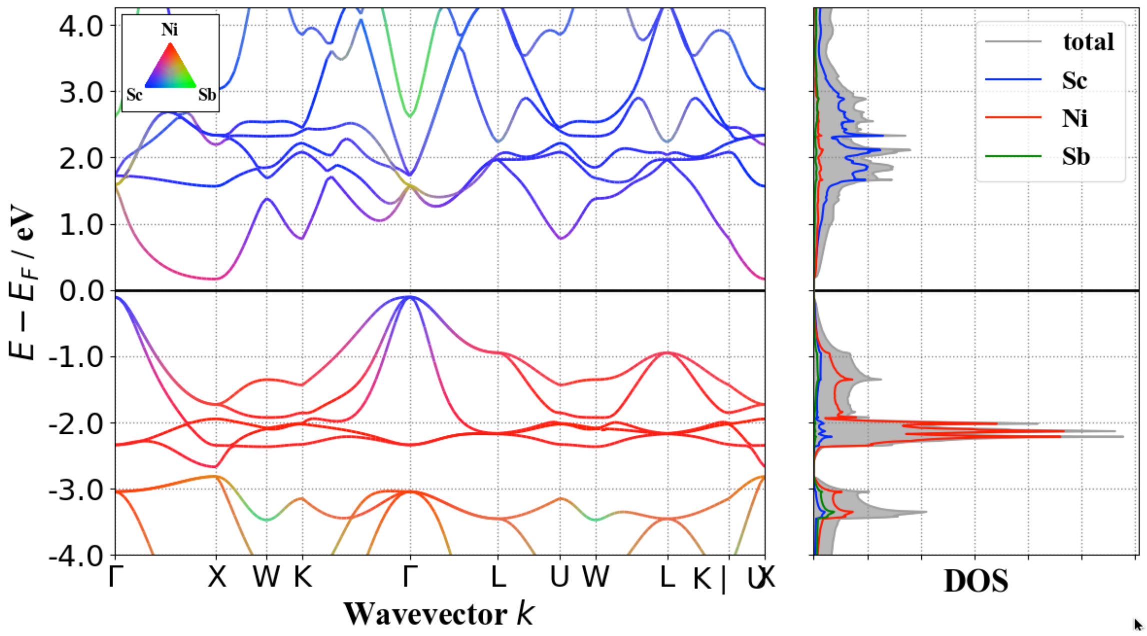

Figure 26: ScNiSb

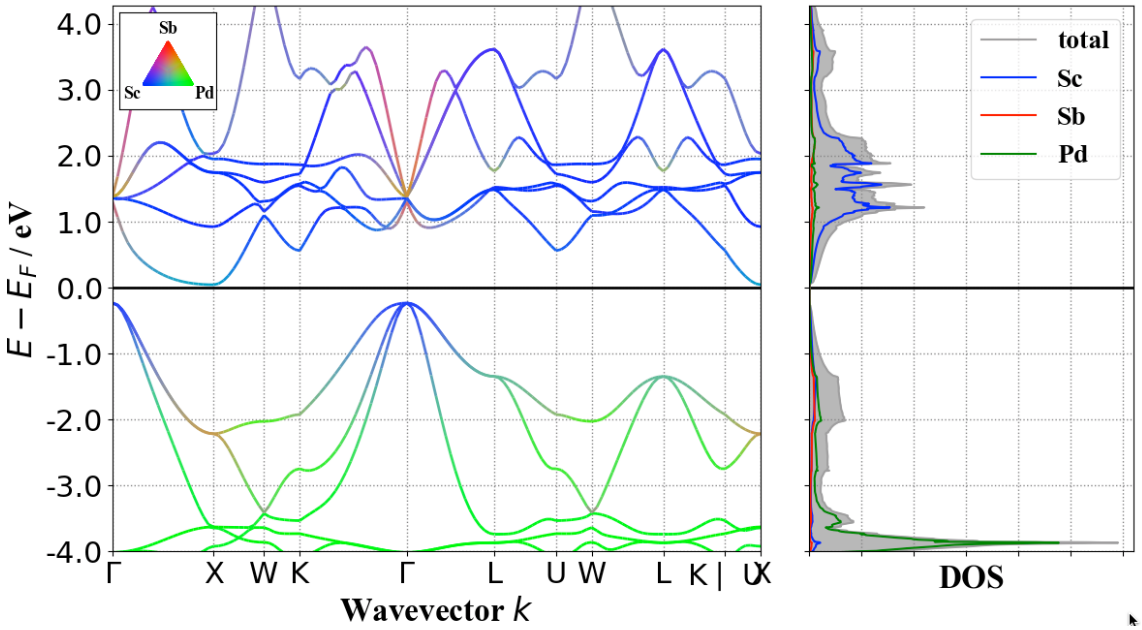

Figure 27: ScSbPd

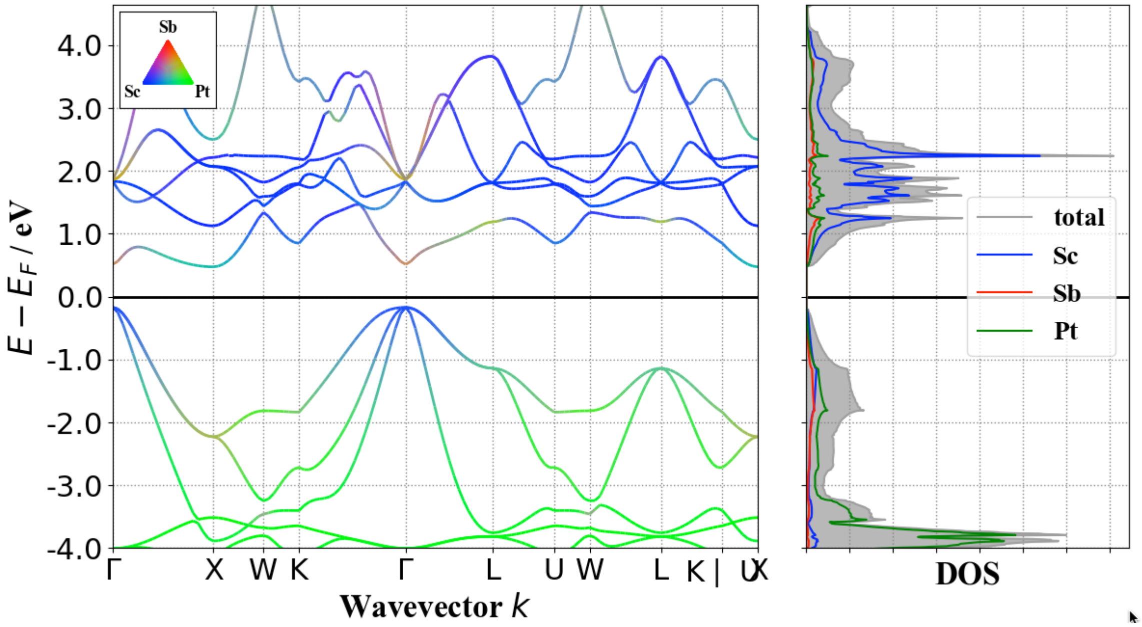

Figure 28: ScSbPt

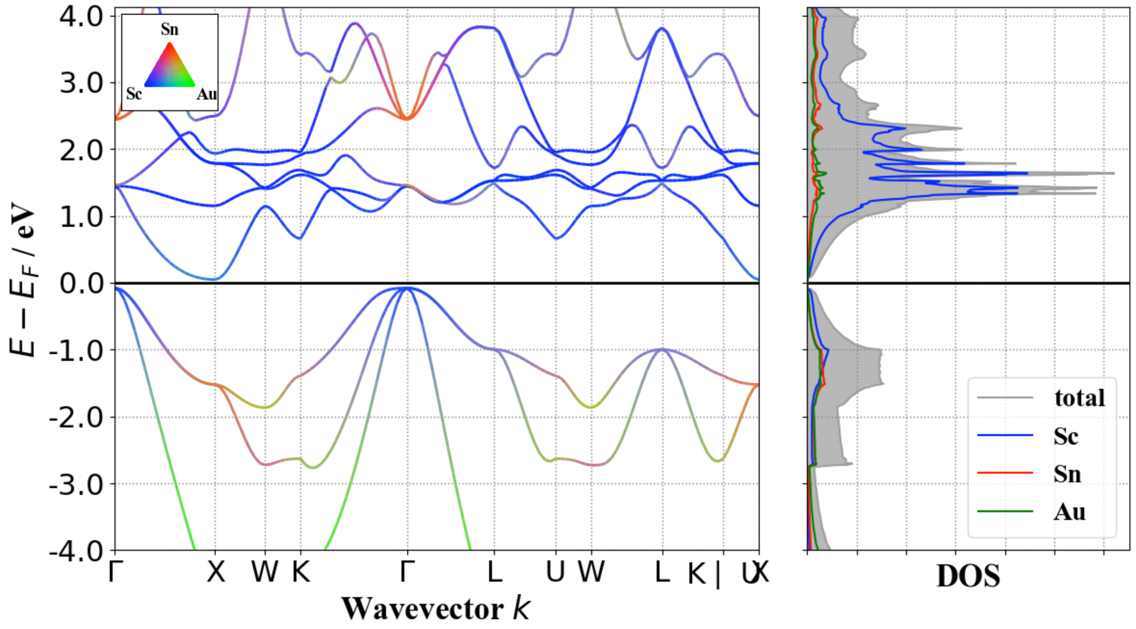

Figure 29: ScSnAu

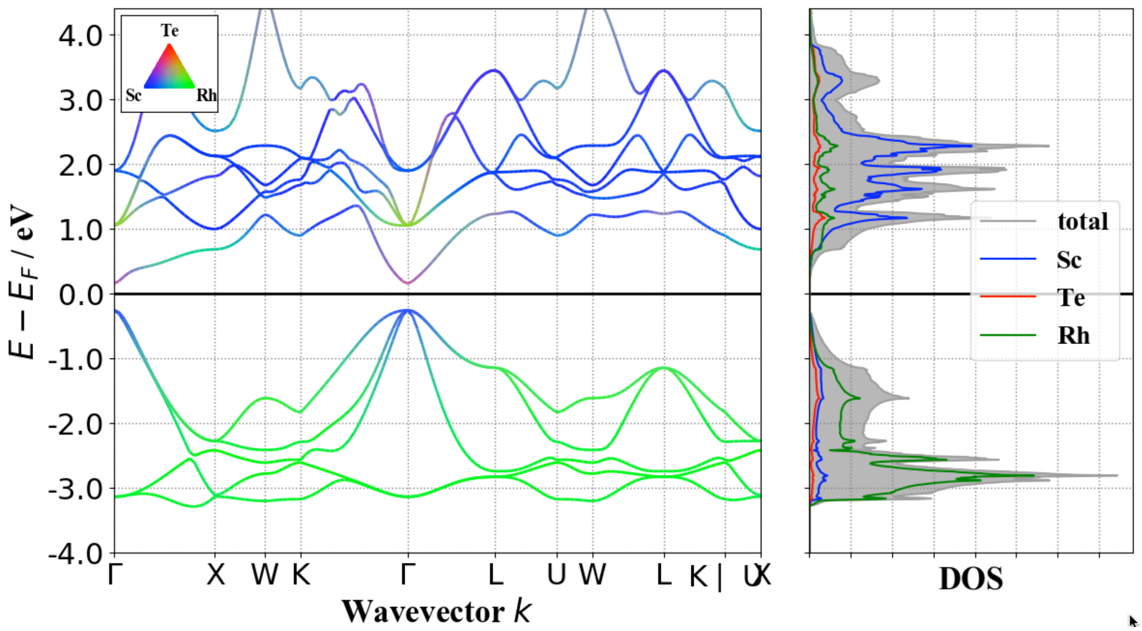

Figure 30: ScTeRh

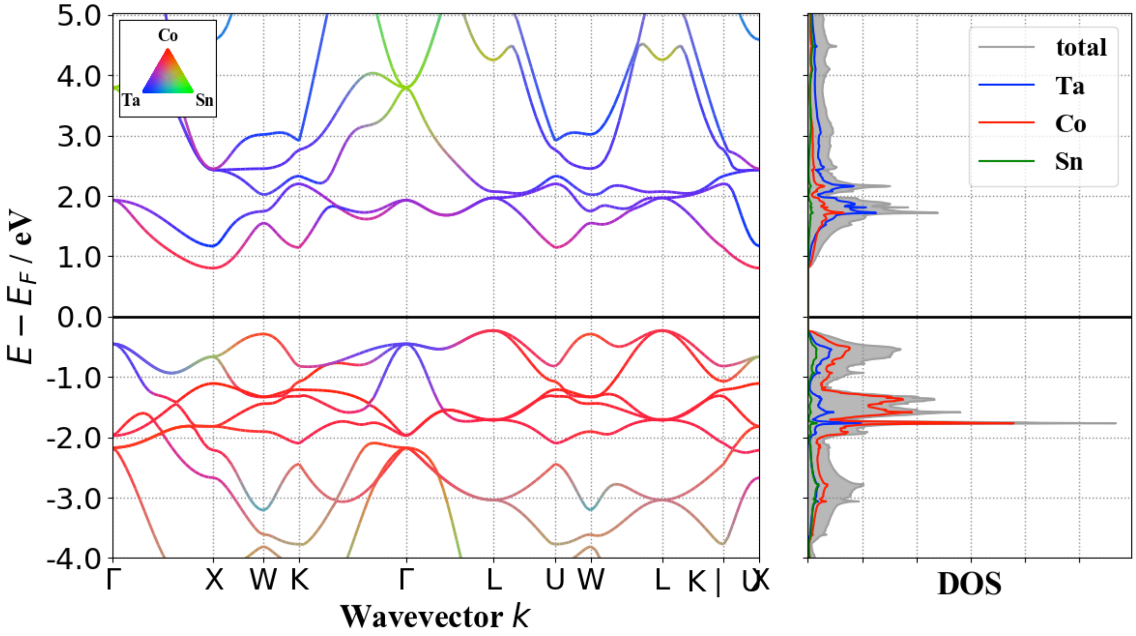

Figure 31: TaCoSn

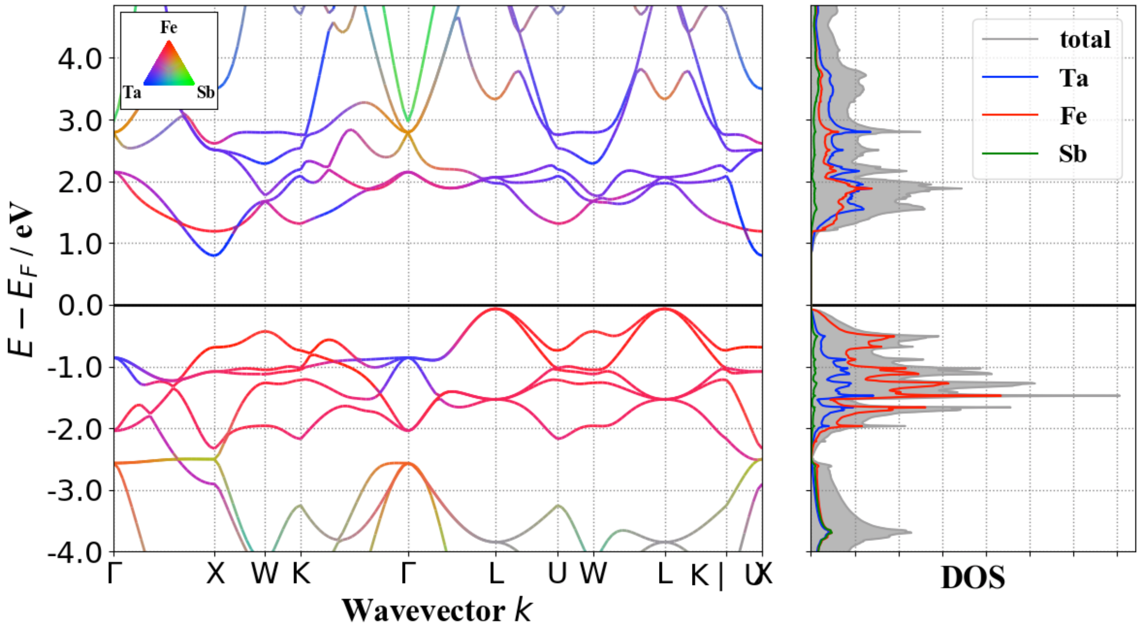

Figure 32: TaFeSb

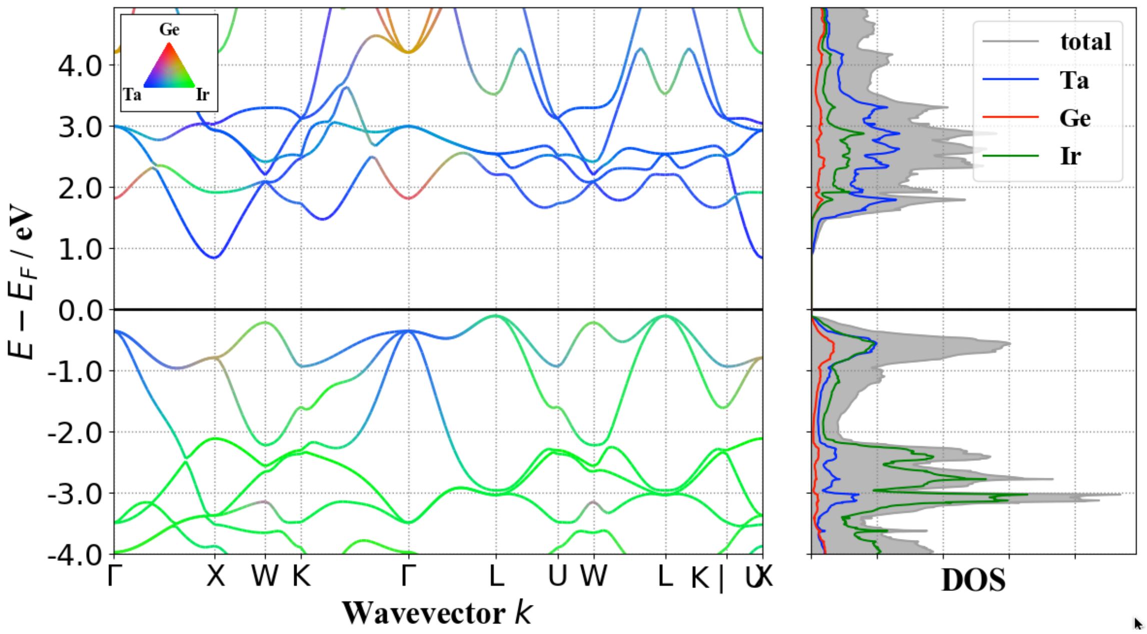

Figure 33: TaGeIr

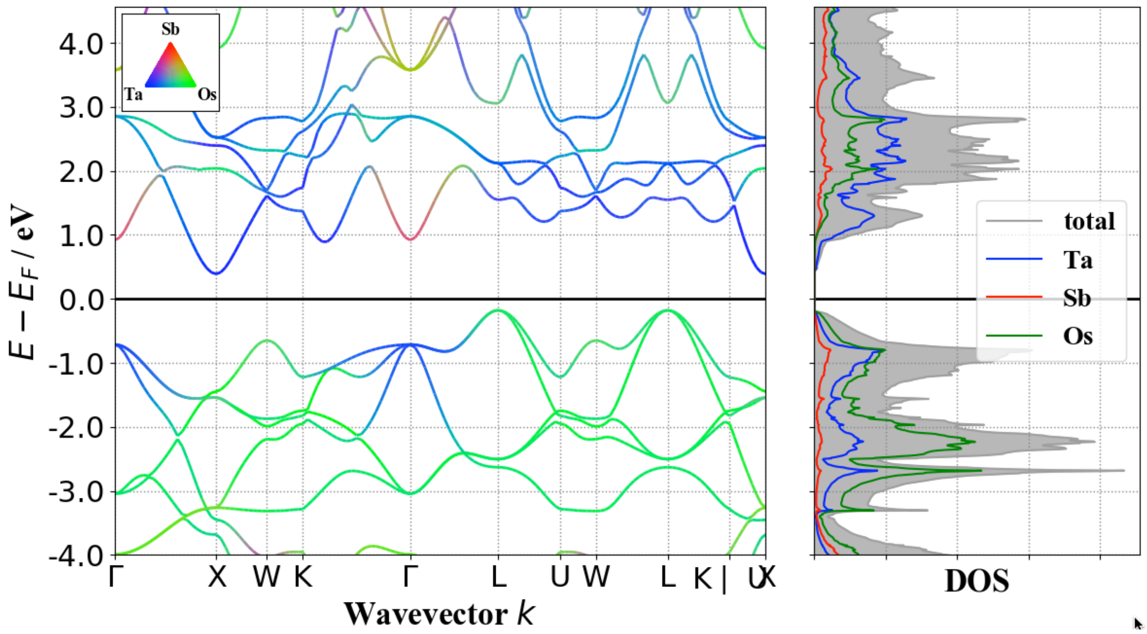

Figure 34: TaSbOs

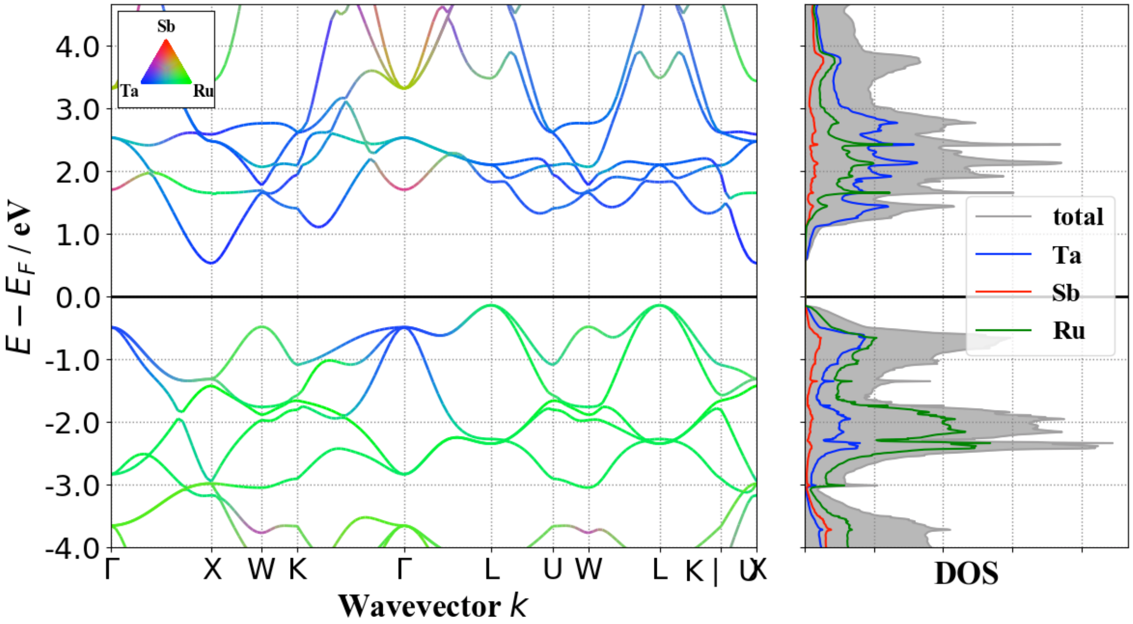

Figure 35: TaSbRu

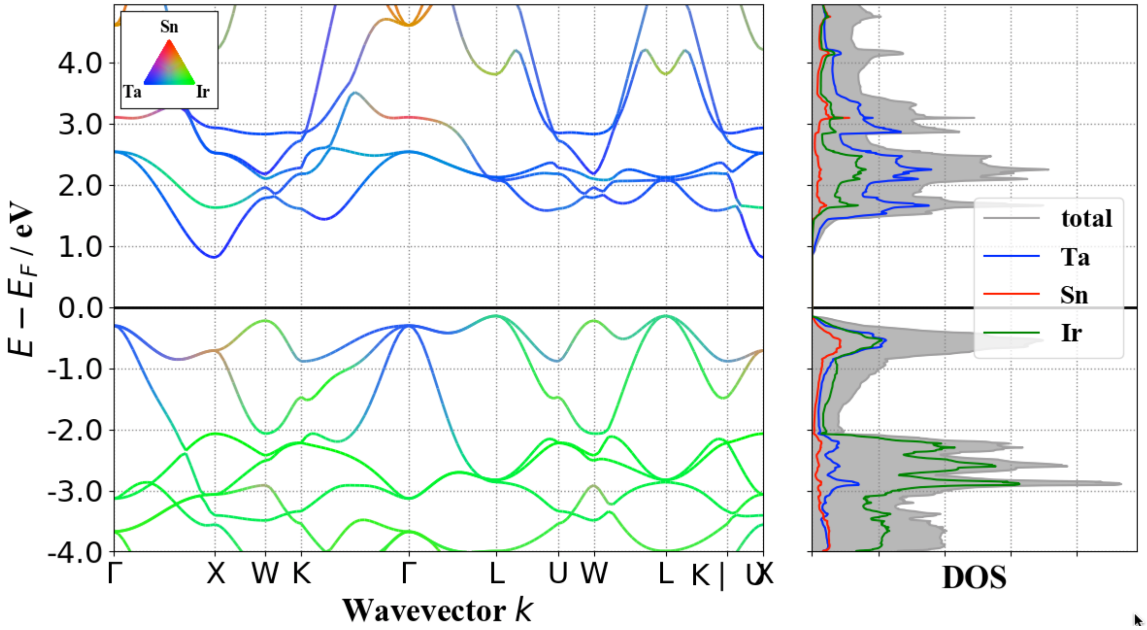

Figure 36: TaSnIr

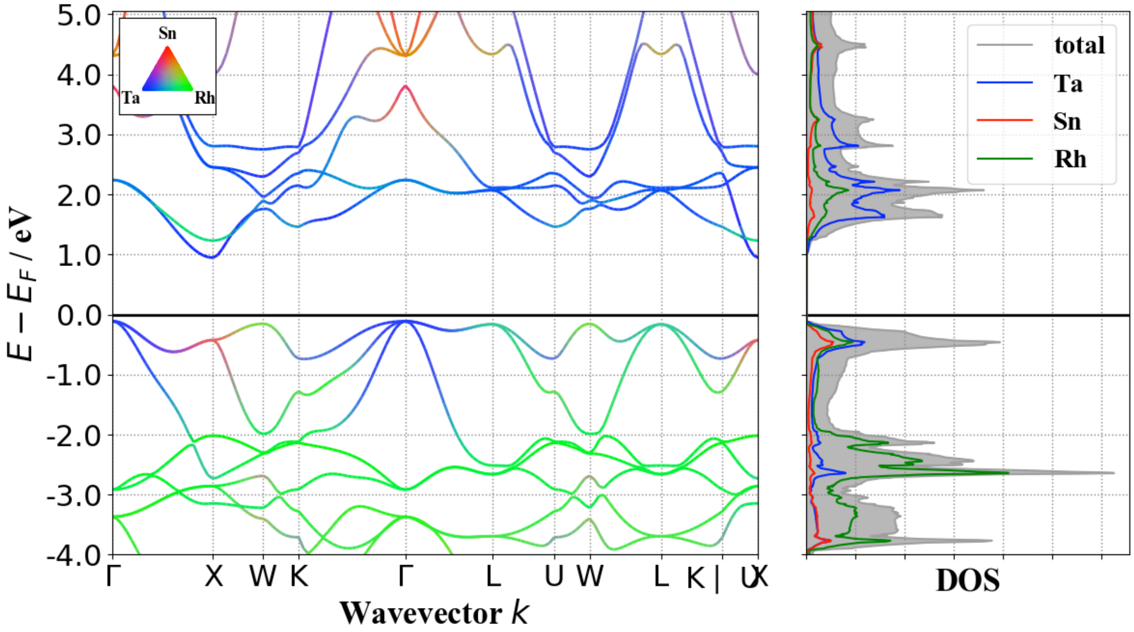

Figure 37: TaSnRh

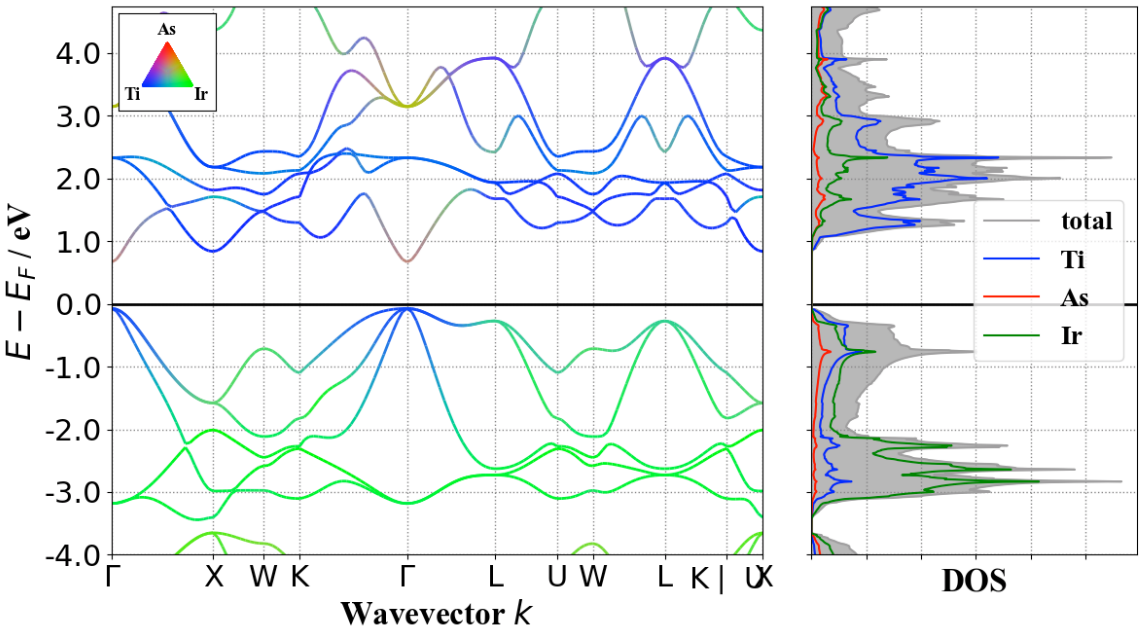

Figure 38: TiAsIr

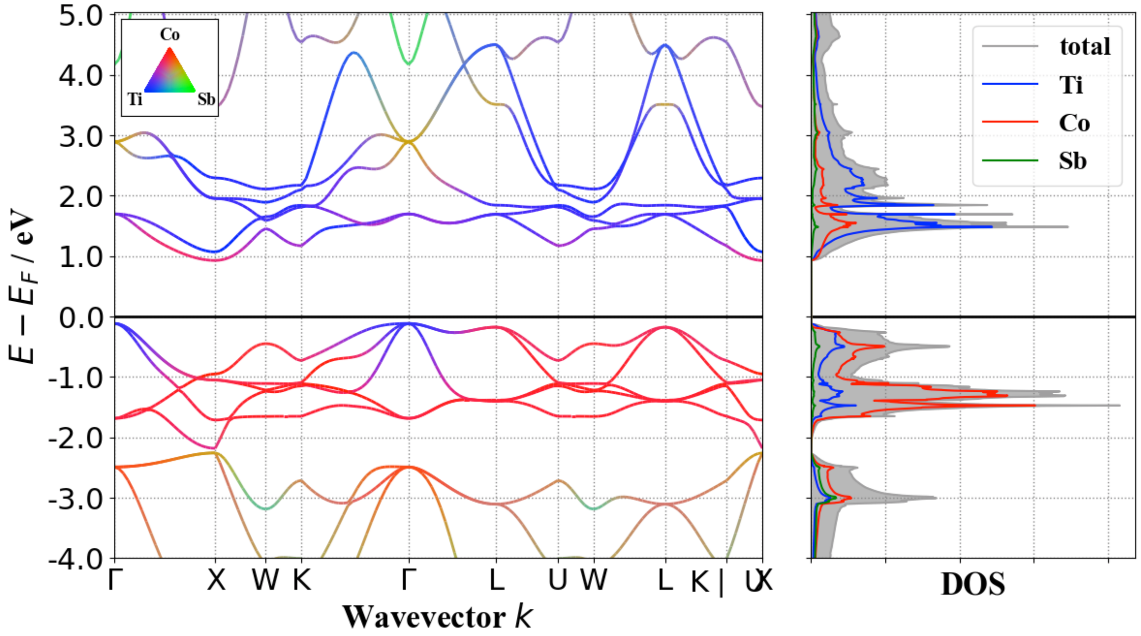

Figure 39: TiCoSb

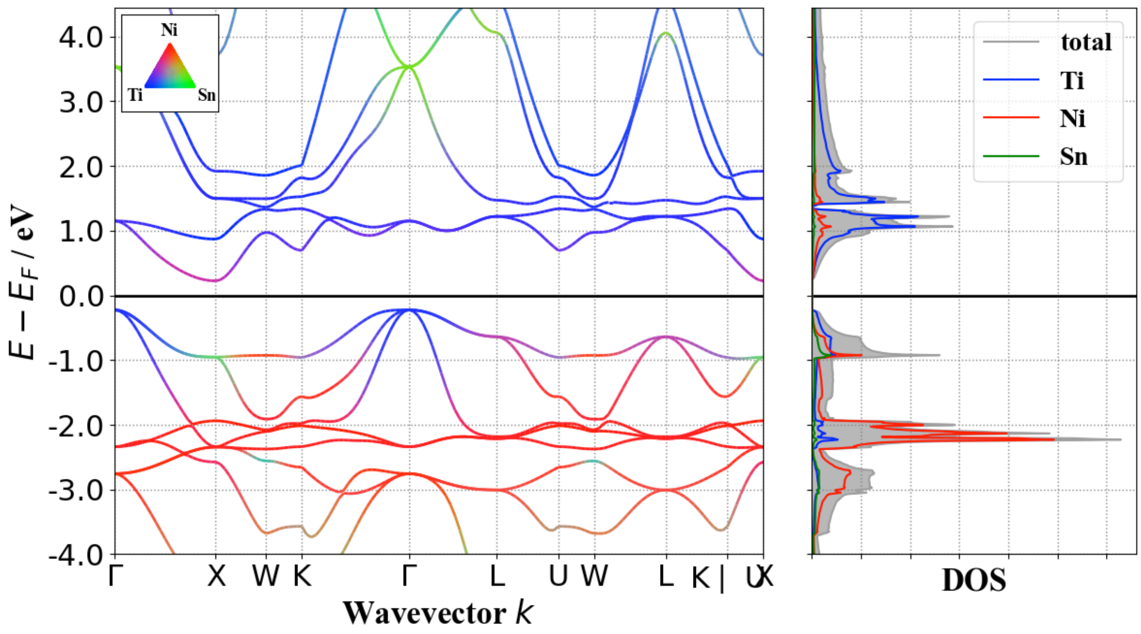

Figure 40: TiNiSn

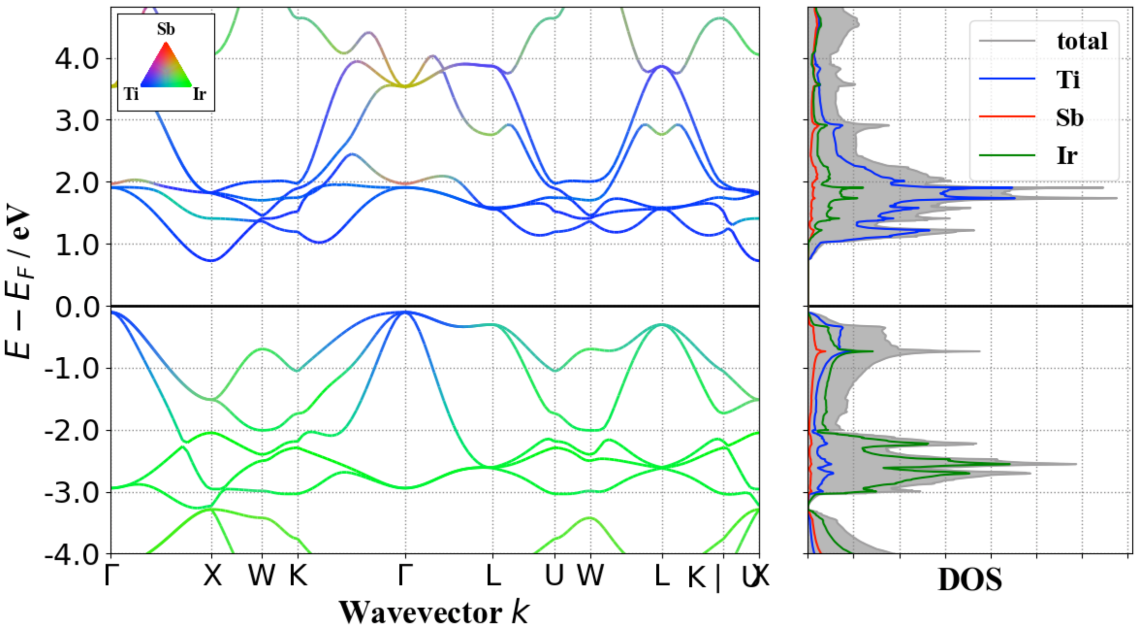

Figure 41: TiSbIr

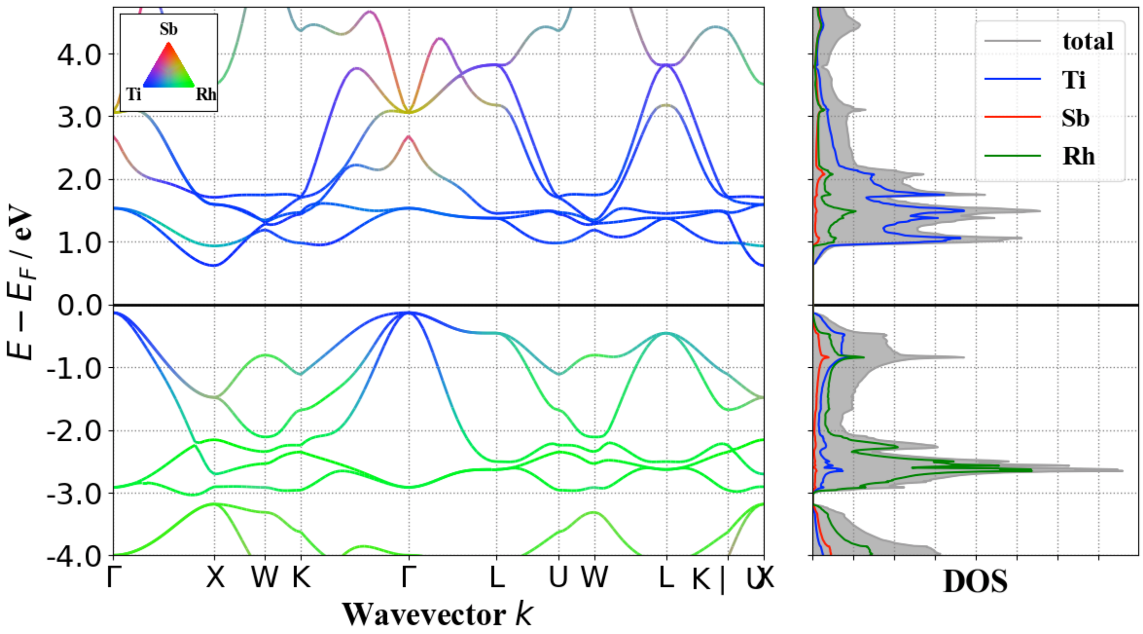

Figure 42: TiSbRh

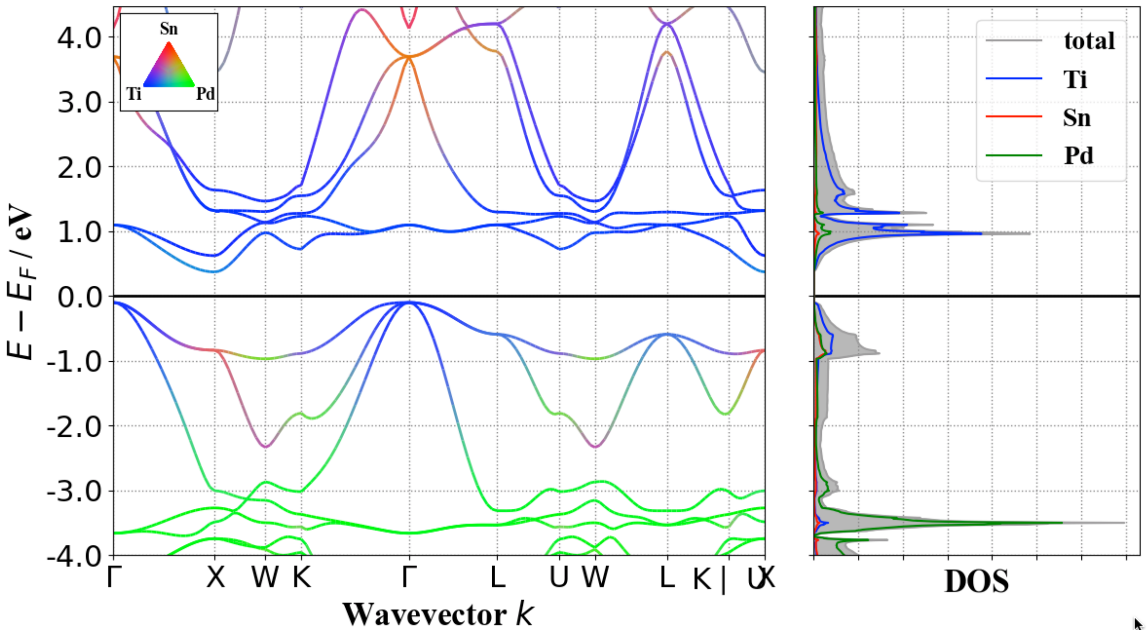

Figure 43: TiSnPd

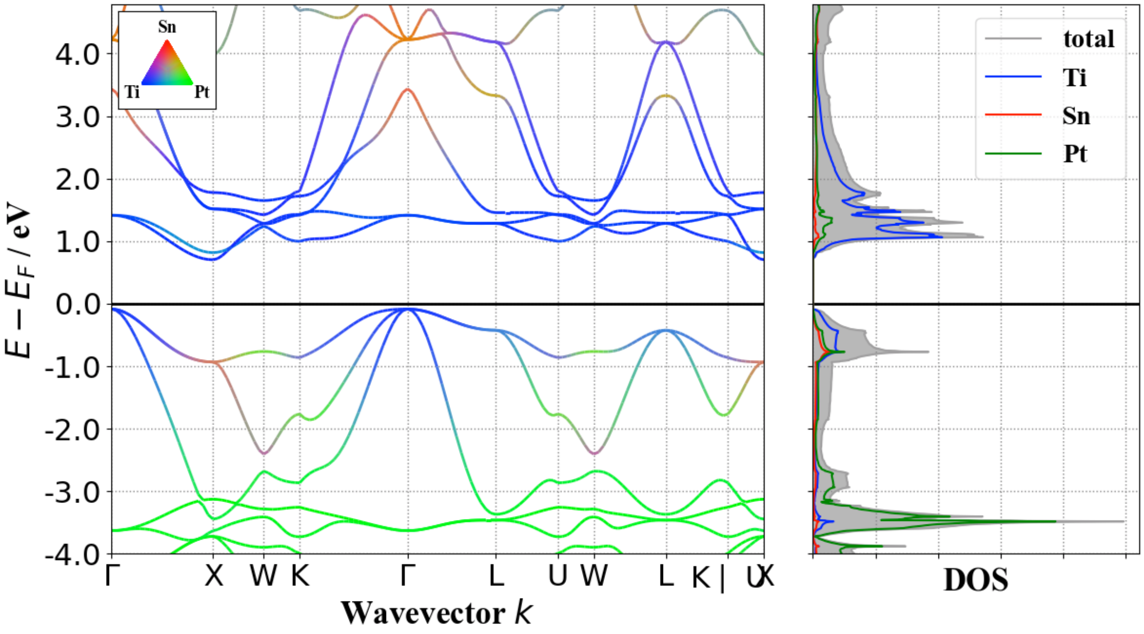

Figure 44: TiSnPt

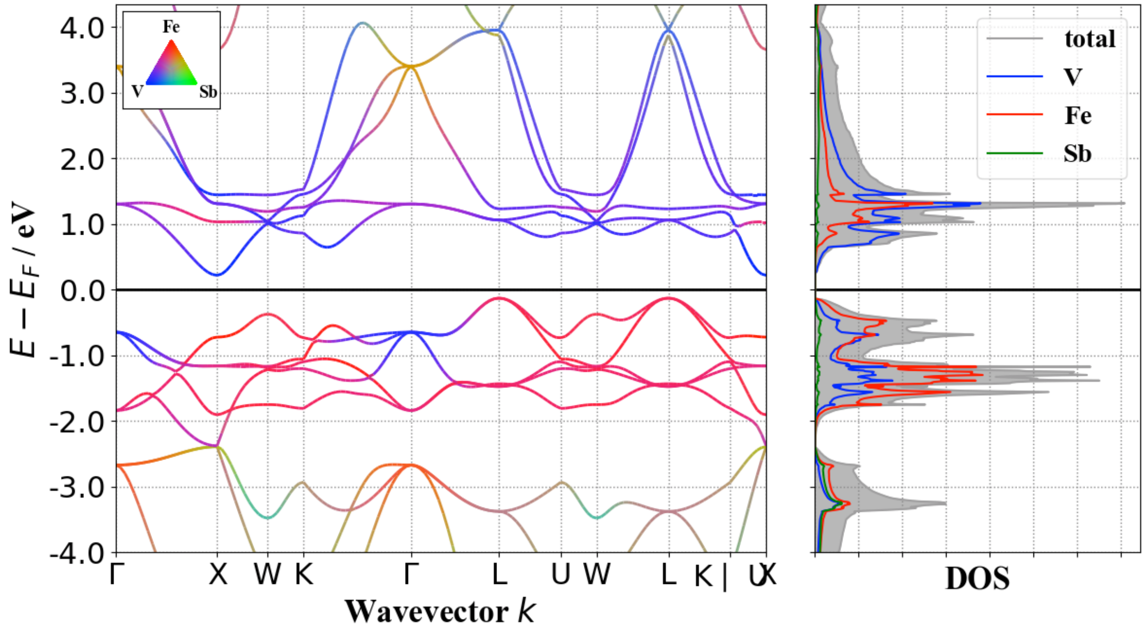

Figure 45: VFeSb

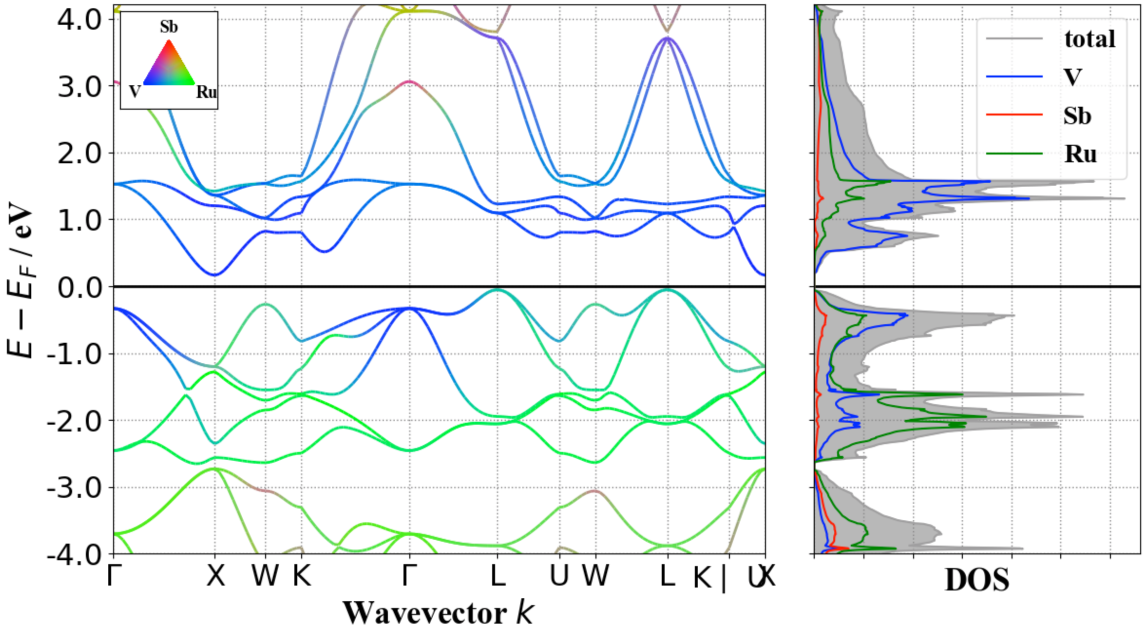

Figure 46: VSbRu

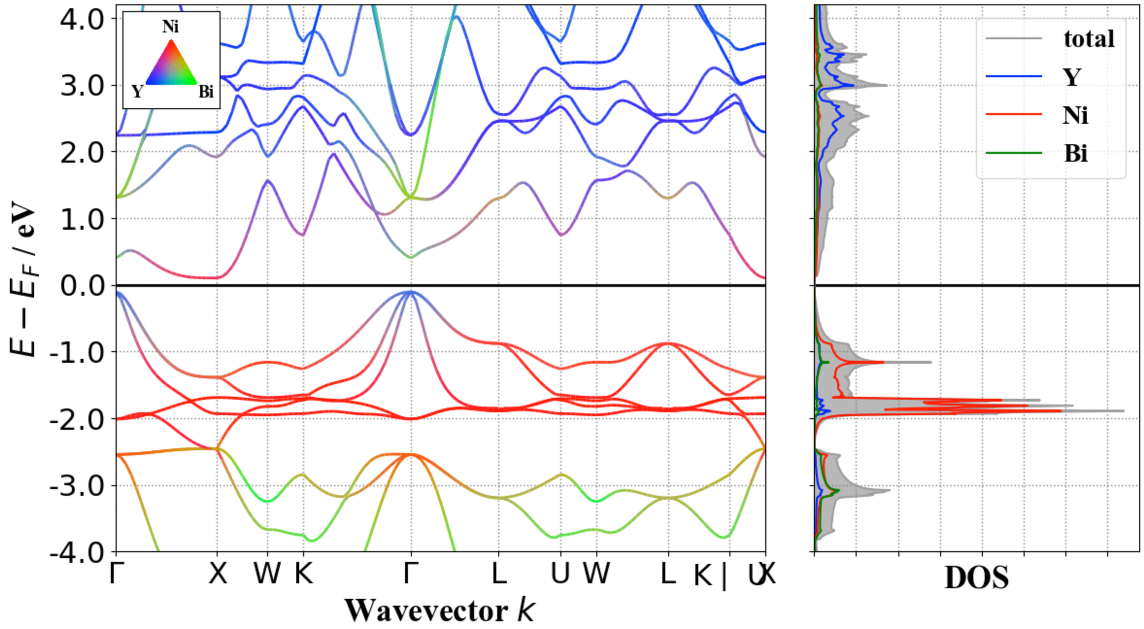

Figure 47: YNiBi

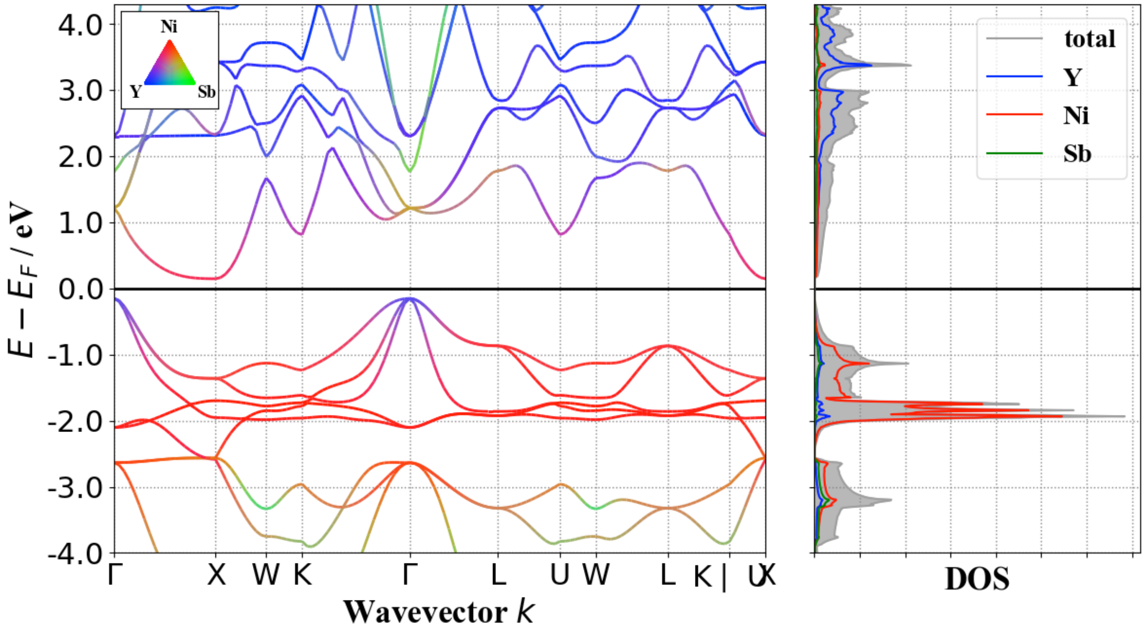

Figure 48: YNiSb

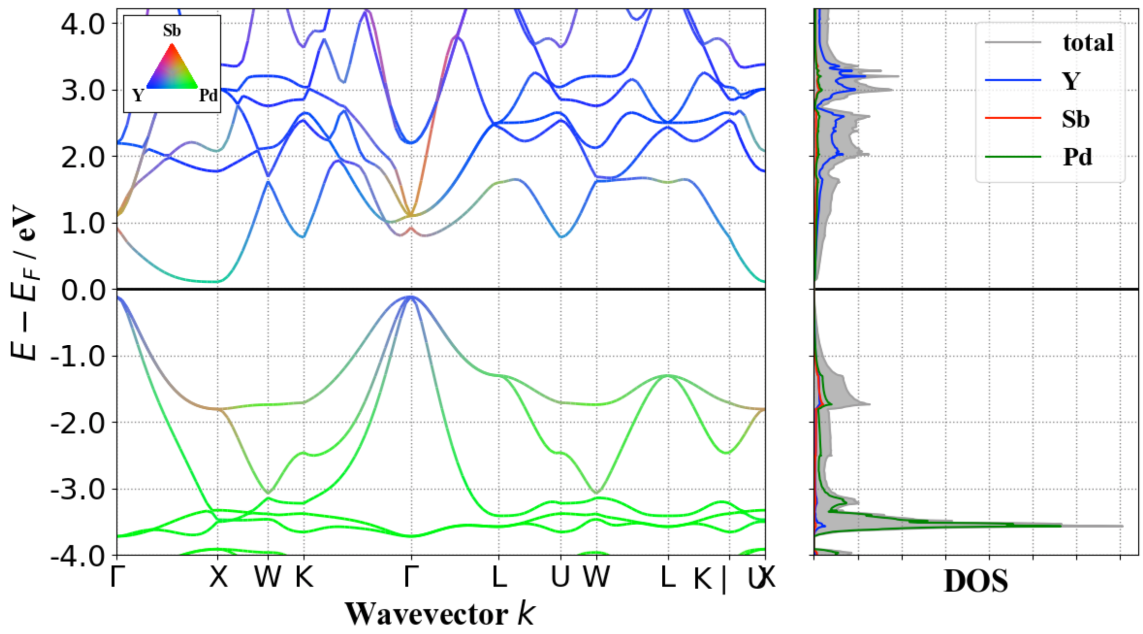

Figure 49: YSbPd

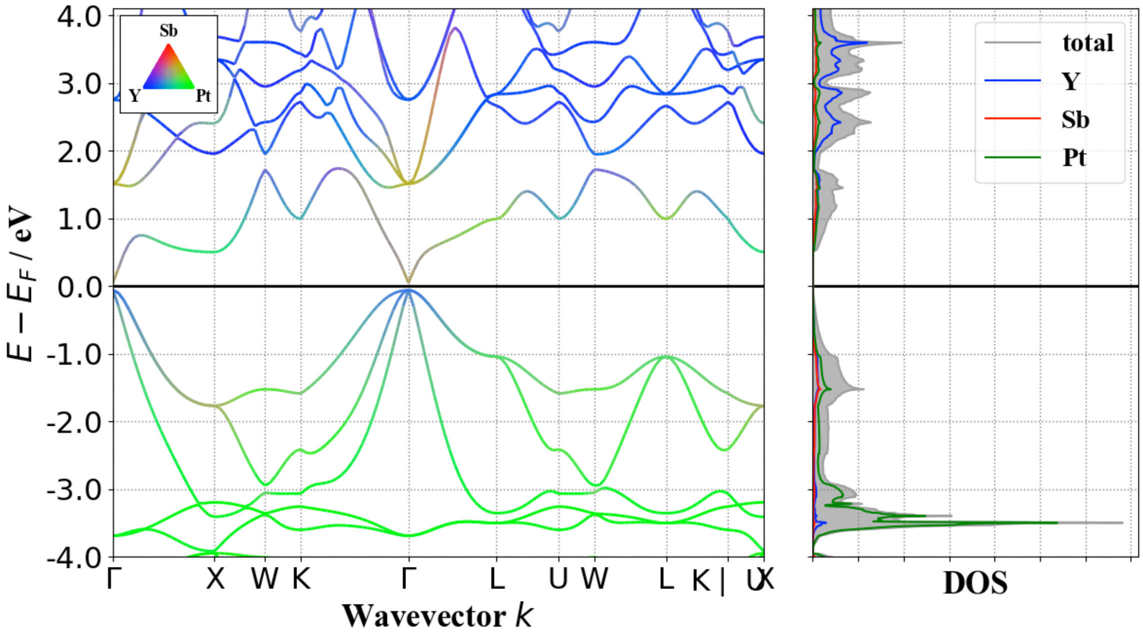

Figure 50: YSbPt

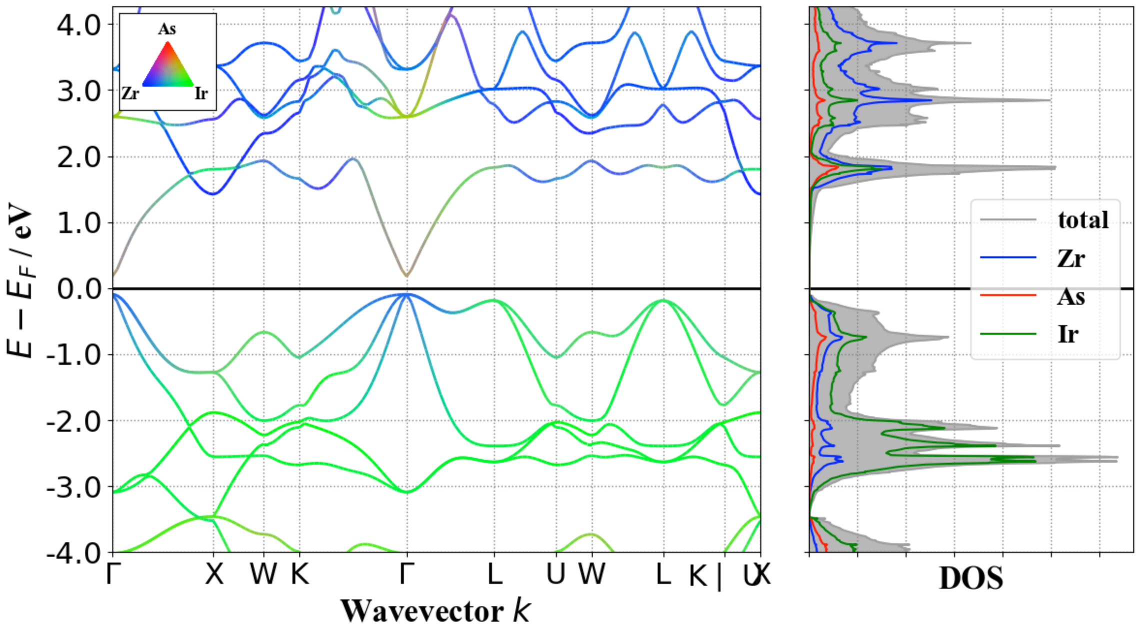

Figure 51: ZrAsIr

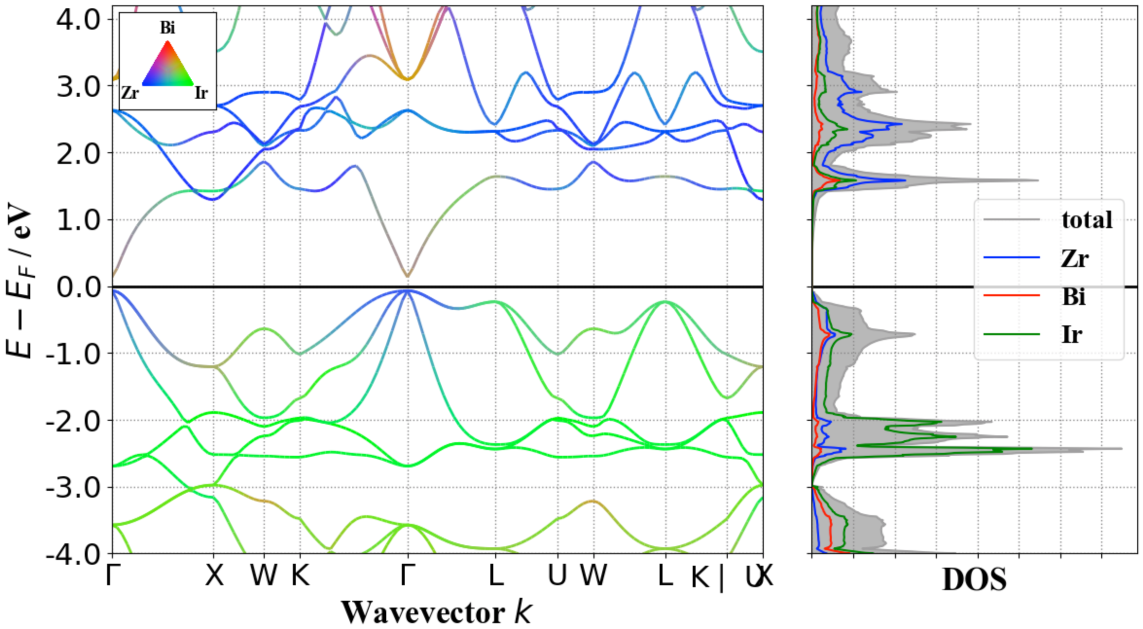

Figure 52: ZrBiIr

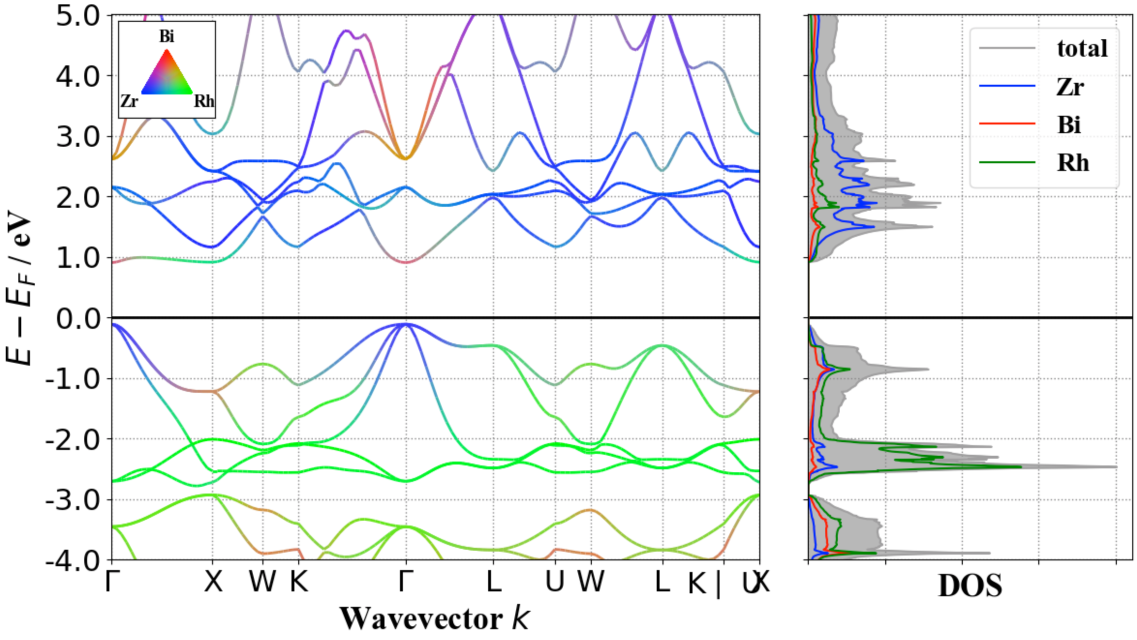

Figure 53: ZrBiRh

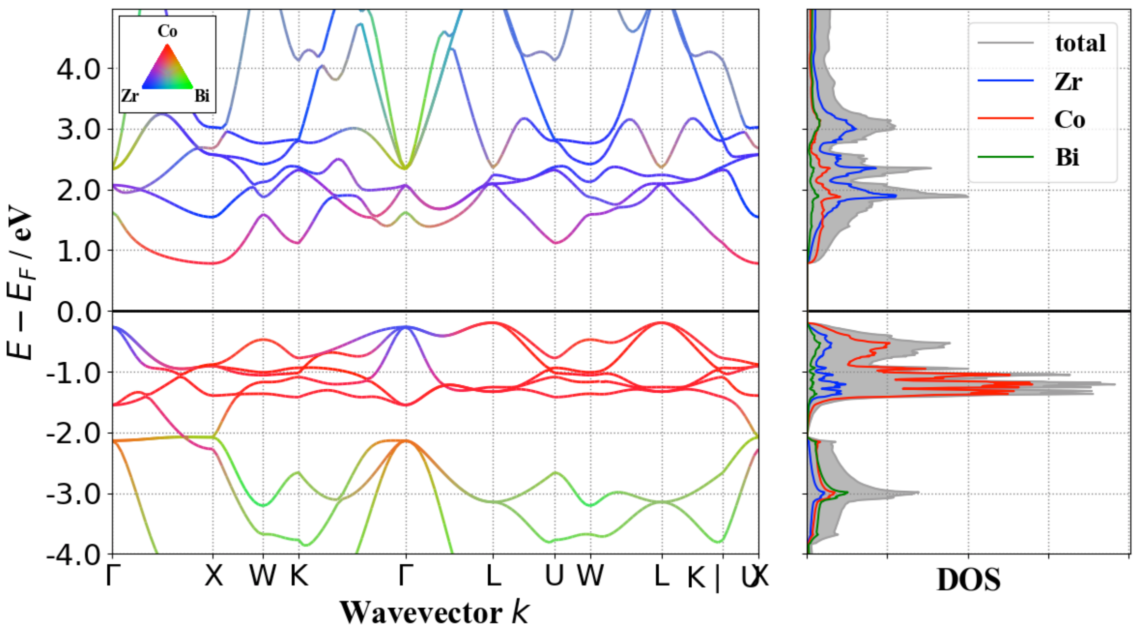

Figure 54: ZrCoBi

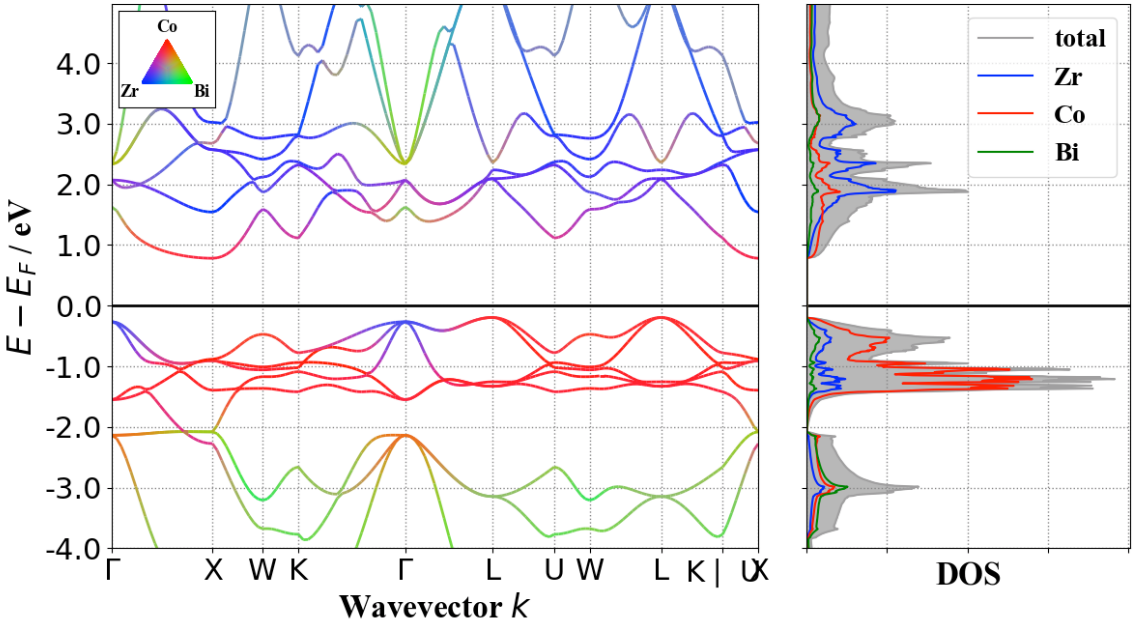

Figure 55: ZrCoBi

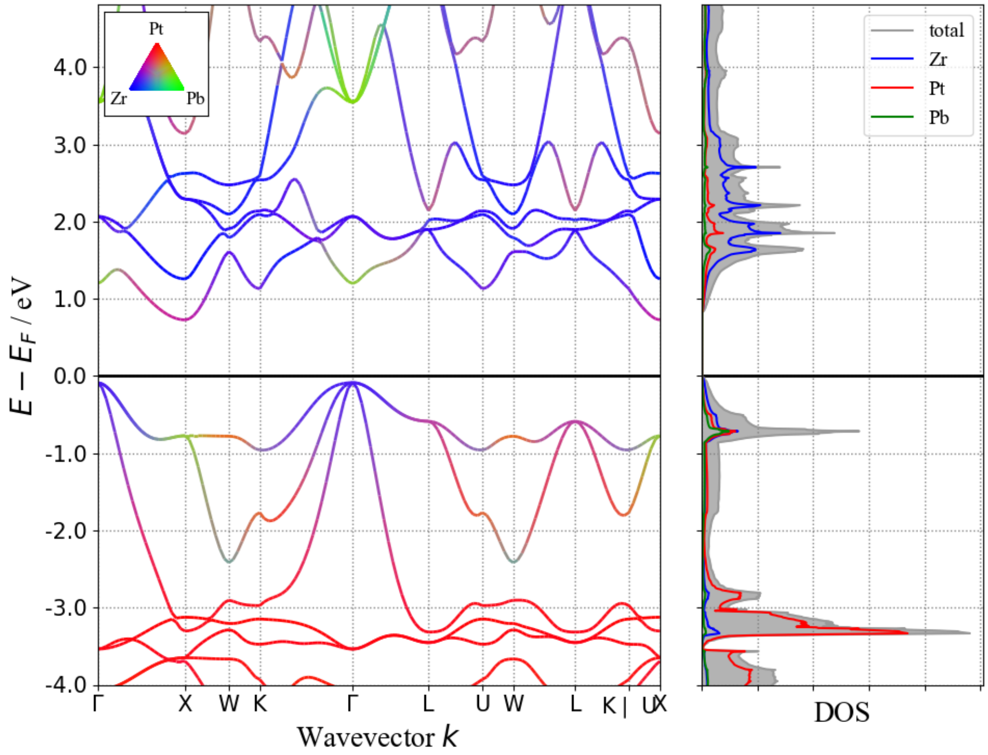

Figure 56: ZrPtPb

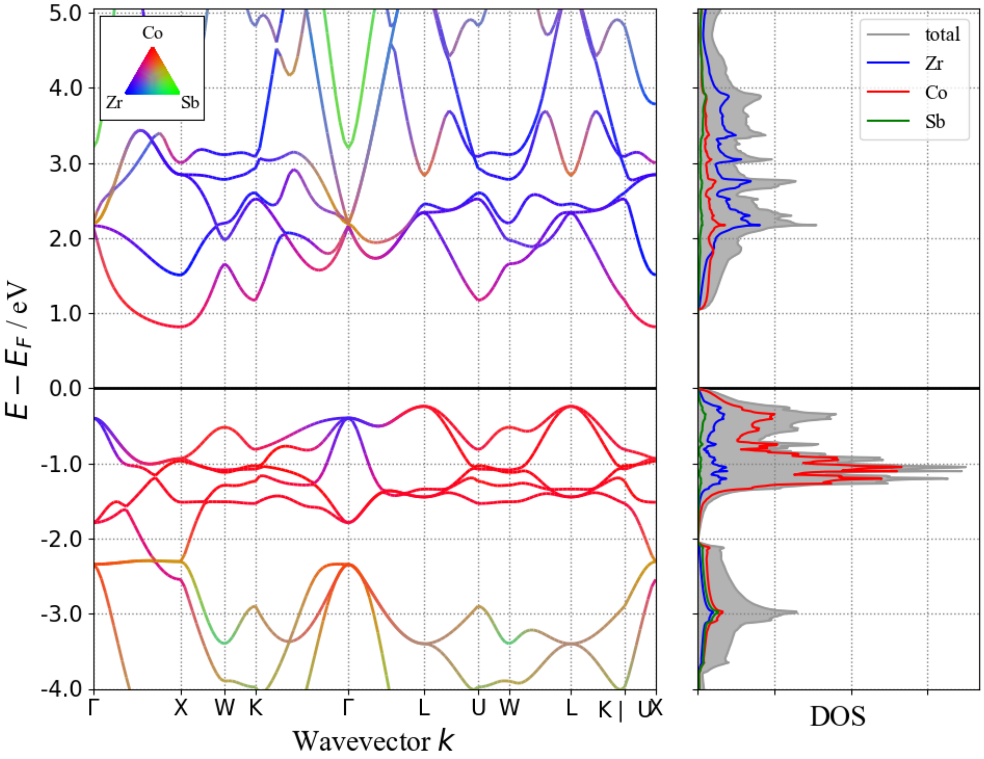

Figure 57: ZrCoSb

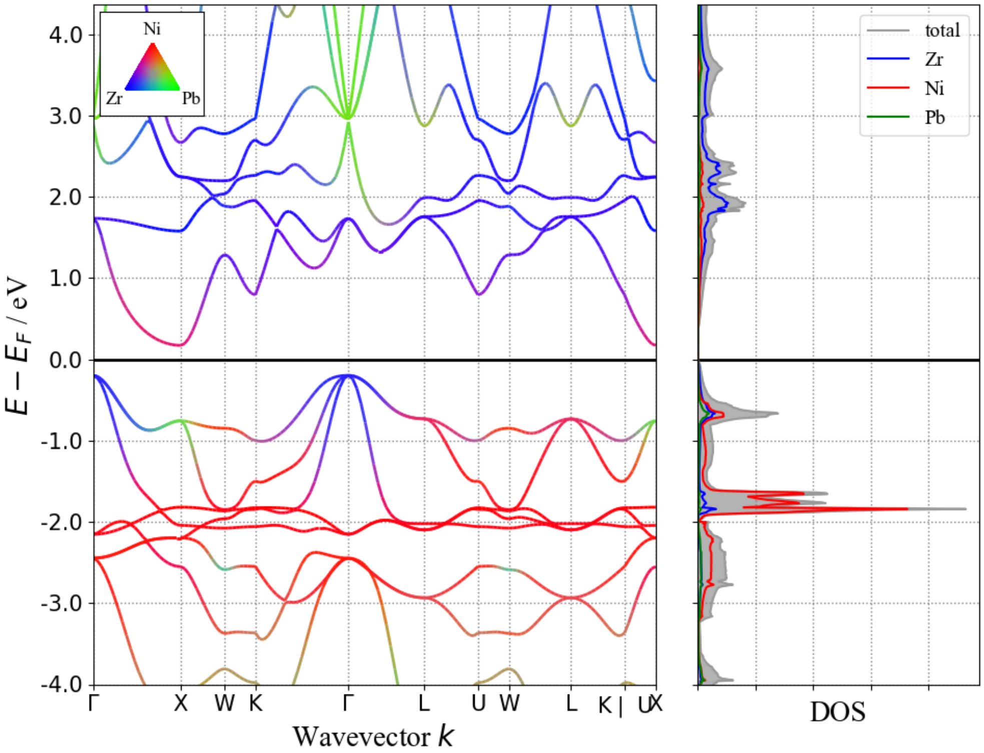

Figure 58: ZrNiPb
